# The Efficacy of Cholesterol-Based Carriers in Drug Delivery

**DOI:** 10.3390/molecules25184330

**Published:** 2020-09-22

**Authors:** Ngonidzashe Ruwizhi, Blessing Atim Aderibigbe

**Affiliations:** Department of Chemistry, University of Fort Hare, Alice Campus, Eastern Cape 5700, South Africa; 201515559@ufh.ac.za

**Keywords:** cholesterol, anticancer, antibacterial, antiviral, wound dressing, drug delivery system

## Abstract

Several researchers have reported the use of cholesterol-based carriers in drug delivery. The presence of cholesterol in cell membranes and its wide distribution in the body has led to it being used in preparing carriers for the delivery of a variety of therapeutic agents such as anticancer, antimalarials and antivirals. These cholesterol-based carriers were designed as micelles, nanoparticles, copolymers, liposomes, etc. and their routes of administration include oral, intravenous and transdermal. The biocompatibility, good bioavailability and biological activity of cholesterol-based carriers make them potent prodrugs. Several in vitro and in vivo studies revealed cholesterol-based carriers potentials in delivering bioactive agents. In this manuscript, a critical review of the efficacy of cholesterol-based carriers is reported.

## 1. Introduction

Drug delivery, which in principle is administering a pharmaceutical compound to achieve a therapeutic effect, continues to gain ground due to the continual rise in the cases of drug resistance [1]. Critical subjects that need careful examination when it comes to drug delivery include the route of drug delivery and the design of the drug delivery system. Drug delivery routes usually used include intravenous, oral, nasal, [2] pulmonary [3], buccal [4], transdermal [5], etc. A drug delivery system can be any device or formulation enabling the introduction of a therapeutic compound into the body. An ideal drug delivery system improves the efficacy and safety of the drug by controlling the time needed for the drug to reach its target organ and the rate of drug release at the site [1].

Nanoparticles [6], micelles [7], liposomes [8], niosomes [9] and polymer–drug conjugates [10] are some of the commonly reported drug delivery systems. The nature of the target organ and the distance from the site of administration determines the type of system that will be used for drug delivery. Furthermore, cholesterol, an important component in cell membranes [11,12,13,14], has been utilized in the development of drug delivery systems. Its efficacy as a major component in drug delivery systems has been reported by several researchers. This review report in vitro and in vivo outcomes of cholesterol-based carriers designed for delivering drugs.

## 2. Cholesterol and Its Function as a Drug Carrier

**Figure 1:**
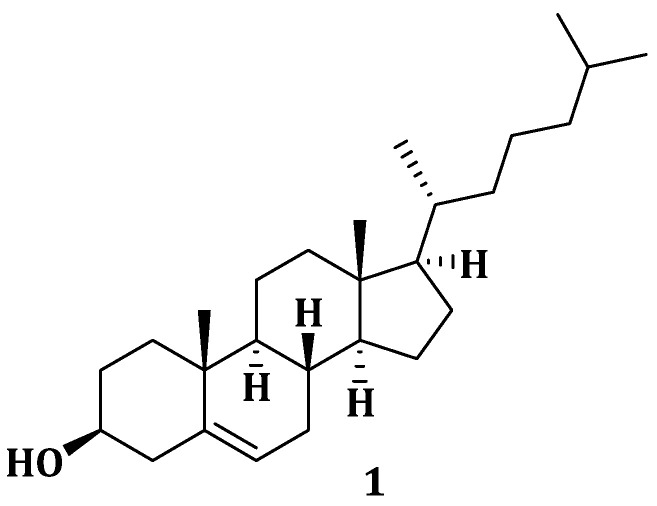
Structure of cholesterol (**1**).

Cholesterol (Figure 1) (**1**) is a fatty substance or a lipid-type molecule that is essential in the human body and also a very important component in the structure of cell membranes [11]. It is a precursor of hormone production [12] (e.g., estrogen and testosterone) and it is used to produce bile, which is essential in aiding digestion. It is required for the function and fluidity maintenance of our nerve cells. It is manufactured in all mammalian cells via the mevalonate pathway but the liver remains the chief organ involved in maintaining cholesterol balance in humans [13]. Its distribution varies in human organs and tissues. It is stocked up in organs such as the adrenals, ovaries and testes as its fatty acid esters, but, above any other organ, the central nervous system (CNS) has the highest amount of cholesterol in the whole body [14]. Due to the wide distribution of cholesterol in the human body, several researchers have reported the use of cholesterol and cholesterol-based carriers in drug delivery [13,14].

The presence of a hydrophilic 3-hydroxy group on cholesterol, together with a hydrophobic hydrocarbon domain, gives it an amphiphilic nature, making it the most recognized sterol [11]. Over the years, several organic syntheses have involved the use of cholesterol as a starting material because it is readily available, affordable and its functional groups are easily derivatized. Many chemical modifications have been performed by reacting known pharmacophores with cholesterol resulting in compounds with enhanced biological activities. Cholesterol-based drug carriers have been designed as delivery systems such as nanoparticles, micelles and liposomal formulations. The presence of cholesterol in a liposomal formulation has been reported to improve the stability of the formulation by preventing the phase transition of phospholipids [15].

Recently, the design of drug delivery systems has taken a new and ever-promising dimension due to the increasing application of cholesterol as one of the materials for the delivering of poorly water-soluble and lipophilic drugs. The unique properties of cholesterol have made it a very attractive candidate for delivering drugs to different organs [16]. These include its good biocompatibility and ability to make membranes stable, easy to functionalize the hydroxyl group, ability to form liposomes that are rigid and promote fusion process, etc. [17]. Linking cholesterol to drugs has also been investigated as a way to avoid irritation of the gastrointestinal tract and poor absorption of orally administered drugs as well as overcoming the first-pass effect [18].

Cholesterol has been successfully used as carriers for different kinds of drugs including antivirals, antimalarials, anticancer, etc. [11]. Similar to any other lipid-based carriers, cholesterol-based carriers can be produced fairly easily in large quantities at low cost, they are biodegradable and biocompatible [19] and they are easily manipulated. Other advantages ascribed to cholesterol-based carriers include high drug loading efficiency [20] and the ability to incorporate lipophilic and hydrophilic drugs [21]. The surface of cholesterol-based carriers can be easily modified. Formulations containing cholesterol was reported to prevent aggregation in the aqueous environment because of their increased hydrophilicity with significant transfection efficiency and serum compatibility [22].

In synthesizing cholesterol-based carriers, different bonds have been used, including ester, amide, hydrazone and disulfide bonds. The ester bond remains the most used bond type. Ester bonds degrade through hydrolysis in the presence of enzymes such as esterases to release the active drugs, if the drug is directly bonded to the carrier. Amide bonds are more stable than ester bonds and this makes them release the drugs slowly. Hydrazone bonds are mostly used to synthesize cholesterol-based carriers which are pH-sensitive. In extracellular oxidative environments, disulfide bonds are stable, but, once in a reductive intracellular environment, they are easily cleaved [18]. Cholesterol-based drug delivery systems such as niosomes do not have a chemical reaction between the non-ionic surfactants and cholesterol but the components are rather mixed in ratios to give dispersions and gels [23,24].

In 2018, Albuquerque et al. reported recent advances in the synthesis and applications of cholesterol-based compounds. In 2014, a review article reported synthetic cholesterol-based compounds through oxidation reactions, substitution on the hydroxyl group, addition on the C5-C6 double bond and functionalizing C-H and C-C bonds. This review is focused on research work which was published between 2015 and 2018 but not reported in the 2018 review paper as well as the latest (2019 and 2020) reports on cholesterol-based carriers in drug delivery.

## 3. Application of Cholesterol-Based Carriers in Anticancer Drug Delivery

In 2018, the World Health Organization (WHO) reported 18.1 million new cancer cases and 9.6 million cancer-related deaths making cancer one of the leading cause of death [25]. The high number of people who die from cancer has prompted researchers to develop several classes of antitumor agents [26]. The effectiveness of most of the currently used anticancer agents is severely limited by drug resistance. Most of the anticancer drugs fail due to drug resistance, thereby making the patients to succumb to the disease [27].

Cholesterol-based carriers have been used in targeted anticancer drug delivery [28]. It accumulates in the ovarian tissue where it is used for the synthesis of sex hormones. This has led to the use of cholesteryl drug conjugates for targeting of the ovary. Research has shown that patients with ovarian tumors had an eightfold uptake of labeled cholesteryl oleate by the tumorous cells compared to the normal ones [13]. Normal cells and cancer cells have differences in substrate uptake. Due to their high uncontrolled growth rate, tumors require more nutrients, which results in the overexpression of various receptors such as the folate, growth factor, transferrin and Low-Density Lipoproteins (LDL) receptors. Investigations on drug delivery systems targeting these receptors have been reported by some researchers [29].

### 3.1. Micelles

Polymeric micelles are promising drug delivery systems which have attracted considerable interest due to their enhanced solubility in water and prolonged blood circulation [30,31]. Incorporation of cholesterol to any drug delivery system improves transportation across the cell membrane. Drugs encapsulated in these micelles can be delivered to specific tissues, prolonging the half-life of the incorporated drug in the blood and achieving a controlled drug release mechanism without exerting severe toxic side effects. Bioactive agents which are incorporated into the core of the polymeric micelles include charged compounds, lipophilic substances and metal complexes. Micelles are structurally stable even at low concentrations [32].

The use of small interfering RNA (siRNA) is a potential therapeutic approach to treat cancer. It acts by silencing genes that contribute to drug resistance during chemotherapy. Its successful therapy is dependent on the use of delivery vehicles due to its biological instability and inefficient cellular uptake [33]. Delivering siRNA to tumor sites is a challenge because it degrades rapidly by nucleases and it is poorly translocated due to its high negative charge. Chemical modification of siRNA has been investigated to overcome its limitations, but it is still characterized by increased non-specific binding and high toxicity. On the other hand, curcumin has long been known to be a hydrophobic chemotherapeutic cancer agent [34]. Muddineti et al. developed a curcumin loaded chitosan-cholesterol micellar system as a potential drug–siRNA carrier for cancer combination therapy [35].

Cellular uptake studies were done by visualizing A549 cells treated with siRNA and curcumin loaded chitosan-cholesterol micelles (C-CCM/siRNA) under a fluorescence microscope. The uptake of the micelles and their accumulation in the cytosol of A549 cells was rapid within 30 min. Thirty minutes after C-CCM and siRNA treatment of A549 cells, the Geo mean fluorescence of A549 was 14,259 ± 246 and 19,153 ± 357, respectively, and it increased to 2245 ± 142 and 3645 ± 168, respectively, after 120 min [35]. The results revealed that the drug uptake from the micelle system was dependent on time. Both fluorescence results showed that the drug-loaded micelles exhibited cellular uptake which was time-dependent. The siRNA/curcumin loaded micelles formulation was stable at 4 °C over a period of one month. The results revealed that micelles prepared from chitosan and cholesterol are promising systems for combination therapy in cancer treatment [35]. However, no cytotoxicity study was investigated to fully understand the micellar system.

**Scheme 1:**
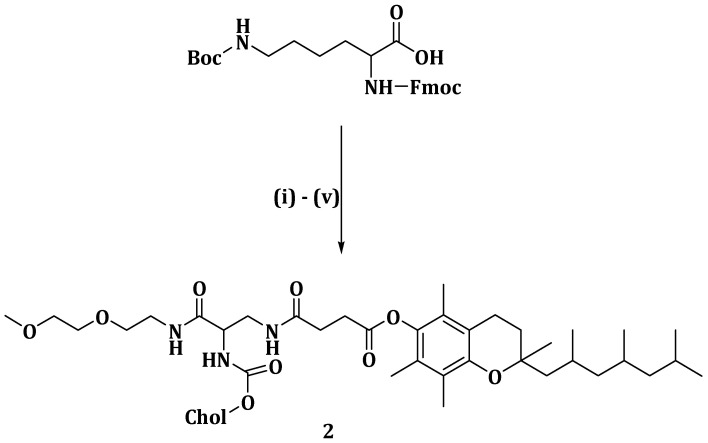
Synthesis of the cholesterol-based amphiphilic polymer (**2**). Reagents and conditions: (i) PEG5K-Amine, EDCl, NHS, TEA; (ii) 4N HCl/Dioxane, 24 h, r.t; (iii) alpha-Tocopherol succinate, EDCl, NHS, TEA, 24 h, r.t.; (iv) piperidine, 4 h, r.t.; and (v) cholesterol chloroformate, TEA, 4 h, r.t.

α-Tocopherol, a form of vitamin E and α-tocopheryl succinate (a-TOS) have also been recently studied as a drug delivery vehicle resulting from their ability to solubilize hydrophobic drugs [36]. α-TOS induce apoptosis in various cancer cells via membrane destabilization and it is a potential multi-drug resistance inhibitor in cancer therapy. Muddineti et al. developed a drug delivery system composed of vitamin E, a lipophilic core of cholesterol and a hydrophilic corona of polyethylene glycol (PEG), covalently bonded by a trifunctional linker, lysine (Scheme 1) (**2**), which was loaded with curcumin [37]. Micelles **2** loaded with curcumin (C-CVM) had a bigger hydrophobic compartment size varying in the range of 162.2–175.8 nm compared to polyethylene glycol amine phosphatidyl ethanolamine micelles (PPM) (20.4–24.4 nm). Micelles **2** displayed high drug encapsulation efficiencies of between 97.2% and 98.6% [37].

It exhibited reduced cell viability, good cellular uptake and sustained drug release in B16F10 and MDAMB-231 cell lines. A low hemolysis activity between 1.10% and 2.81%, which is below the acceptable range of less than 5%, was reported in the cholesterol vitamin E-based polymer, indicating the safe use of the formulation for in vivo studies. Cellular uptake studies showed that the micelles, **2** and C-PPM were taken up in dose- and time-dependent manner as the fluorescence was brighter after 4 h when compared to an hour after administration of the formulation [37]. Both micelles, **2** and C-PPM, showed no significant difference in the cellular uptake which is due to the presence of PEG-based polymeric corona. In vitro cytotoxicity evaluation on the free curcumin, CVM, PPM and micelles **2** and C-PPM in B16F10 and MDA-MB-231 cell lines revealed that PPM showed no cytotoxic effect on both cell lines with nearly 100% cell survival. However, the presence of α-TOS led to CVM showing significant cytotoxic effect [37].

Cytotoxic study profiles of C-PPM, free curcumin and micelles **2** for 6 h followed by incubation for 24 h were 48.7%, 58.2% and 42.8, respectively, in B16F10 cells and 74.2%, 59.6% and 58.2%, respectively, in MDA-MB-231 cells. The cell viabilities demonstrated by CVM and PPM (at a concentration of 50 µg/mL) were 42.4% and 91.7%, respectively, in B16F10, 45.2% and 92.3% in MDA-MB-231 cells, respectively. The results showed significant cytotoxicity induced by micelles **2** and this was due to the synergism between α-TOS-conjugated CVM and curcumin. Growth inhibition results showed that the cellular uptake of micelles **2** into the tumor spheroids inhibited cell proliferation resulting in an enhanced therapeutic effect. The developed micelles **2** are potential therapeutics for the treatment of drug-resistant tumors [37].

**Figure 2:**
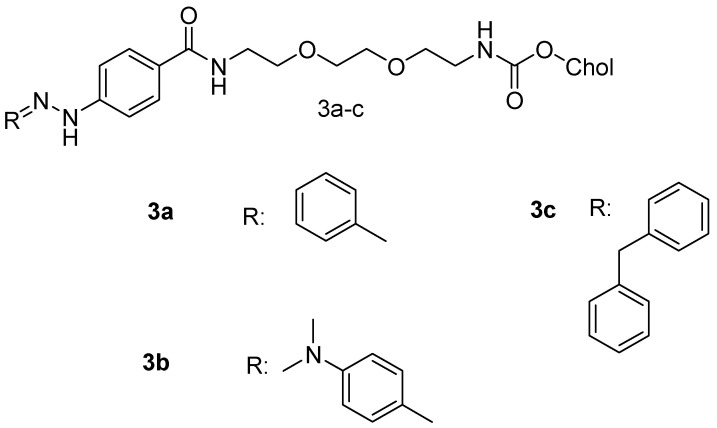
Chemical structures of cholesterol-based hydrazone tethered amphiphiles, compounds **3a**–**c**.

Sarkar et al. synthesized cholesterol-based hydrazone (CBH) tethered tiny amphiphiles with different carbonyl residues (Figure 2) such as benzaldehyde (3a), *p*-dimethylaminobenzaldehyde (3b) and benzophenone (3c). Compound **3a** was the most stable of the three and its drug loading efficiency of doxorubicin (DOX) was 57%. Drug release studies of the DOX-loaded compound **3a** showed that below the pH of 6.5, the drug-loaded vesicles released DOX better than at pH 7.0–8.0, possibly due to the hydrazone bond cleavage [38]. The release pattern is important since most tumor cells have both their intracellular and extracellular pH below 6.5 [17], meaning the anticancer drug is released right at the site of action enhancing its efficacy.

### 3.2. Nanoparticles

**Scheme 2:**
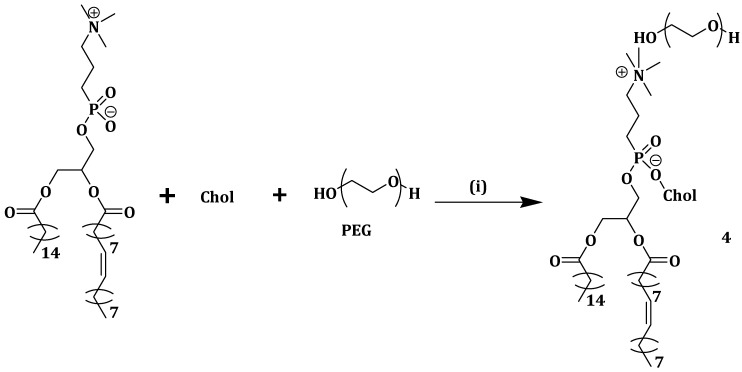
Synthetic route for liposomes containing lecithin, PEG and cholesterol (**4**). Reagents and conditions: (i) soy lecithin, chol, ethanol, stir, 20 min, PEG, stir, 24 h, 25 °C.

Liposomes have been used as a drug delivery system but suffer from drawbacks such as high leakage of the encapsulated drugs, high production cost, fast clearance from circulation and short stability [39]. Heidari et al. prepared titania nanotubes (TNTs) in which 5-fluorouracil, a chemotherapeutic drug, was filled into the cylindrical empty space. Liposomes made of soy lecithin and cholesterol (Scheme 2) were used to coat the drug-loaded TNTs capping the nanotube hole to prevent drug leakage. The drug loading was 100% in the nanotubes. The rate of release of 5-fluorouracil was influenced by the liposomal layers coating on the surface of the nanotubes. At TNT concentrations of <300 μg/mL and between 300 and 1500 μg/mL, about 90% and 80% of the HeLa cells were alive, respectively [40]. The highest toxicity was noted above 3000 μg/mL [41]. Cytotoxicity studies revealed that 5-fluorouracil loaded with compound **4** had a decreased IC_50_ of 250 μg/mL when compared to about 470 μg/mL for the free 5-fluorouracil. Compound **4** displayed enhanced cellular internalization with effective delivery of 5-fluorouracil into the cells [40].

DOX can be readily loaded into DNA nanoparticles for drug delivery [42]. Choi et al. synthesized amphiphilic DNA-cholesterol/DNA-peptide hybrid duplex that assembled into DNA nanoparticles (c-DNA-p nanoparticles) in an aqueous solution. The binding efficiency studies were done by analyzing the fluorescent bands of free DOX and DOX/c-DNA-p complexes on 2% agarose gel electrophoresis. That 0.5 mM of the nanoparticles fully complexed with 3.5 mM DOX revealed the absence of free DOX, indicating a simple and efficient method of loading DOX into the nanoparticles. DOX was fully bound to c-DNA-p nanoparticles at neutral pH 7.4 but the drug began to dissociate from the DOX/c-DNA-p complex at acidic pH of 6.5. At pH 5.0, most of the DOX dissociated from the complex thus indicating the lowering of the binding affinity between DOX and the c-DNA-p nanoparticles which could facilitate cytosolic DOX release [43].

Nanoparticle instability and limited drug loading capacity in most cholesterol-containing polymeric nanoparticles lead to premature drug release into the plasma before reaching the tumor sites. Gonzalez-Fajardo et al. designed and synthesized a drug vehicle, P(NBCh9-bNBPEG), with several cholesteric and PEG side chains as hydrophobic and hydrophilic blocks, respectively. The brush-like architecture had the ability to form stable nanoparticles for the encapsulation of hydrophobic drugs [44]. DOX was loaded into the P(NBCh9-b-NBPEG) nanoparticles (DOX-NPs) and in vivo studies were performed in a xenograft mouse tumor model. There was a significantly reduced tumor growth without side effects when the mice were treated with DOX-NPs. The low release of DOX from the nanoparticles in the blood as well as the reduced accumulation in the major organs like liver, spleen and heart revealed the improved safety profile of DOX-NPs when compared to the free drugs. The results revealed the potential of P(NBCh9-b-NBPEG) nanoparticles for the delivery of hydrophobic drugs with reduced toxicity [44].

**Figure 3:**
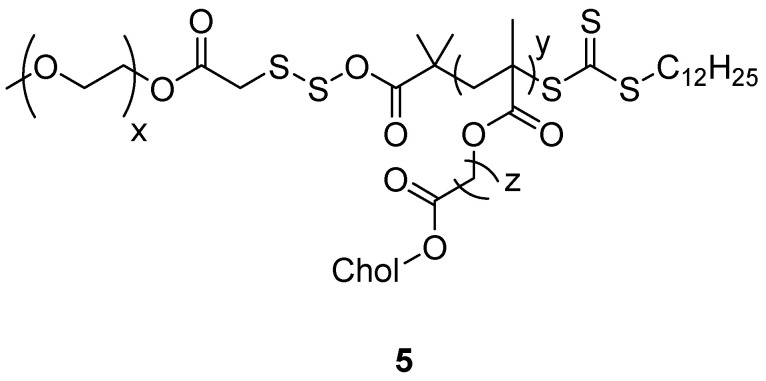
Structure of cholesterol-based block copolymer with disulfide linkage (**5**).

Nguyen et al. synthesized a novel amphiphilic cholesterol-based block polymer comprising of polymethacrylate with cholesterol and PEG having reducible disulfide bonds (PC5MA-SS-PEO) (Figure 3) (**5**) as a nanoparticulate redox-sensitive delivery system. DOX was encapsulated to the self-assembled nanoparticles and the drug loading was 18% *w*/*w* with 95% drug encapsulation efficiency. The DOX-loaded nanoparticles exhibited excellent stability and readily released DOX in dithiothreitol reductive conditions. Notably, the reducible nanoparticles **5** rapidly released DOX inside the tumor cells thereby inducing higher toxicity when compared to the non-reducible PC5MA-PEO-thioester nanoparticles. The in vivo images taken from A549 tumor-bearing severe combined immunodeficiency mice showed that the nanoparticles **5** accumulated preferably in the tumor tissues when compared to the healthy cells/tissues revealing that they are promising delivery systems for the delivery of anticancer drugs with reduced toxicity [45].

**Scheme 3:**
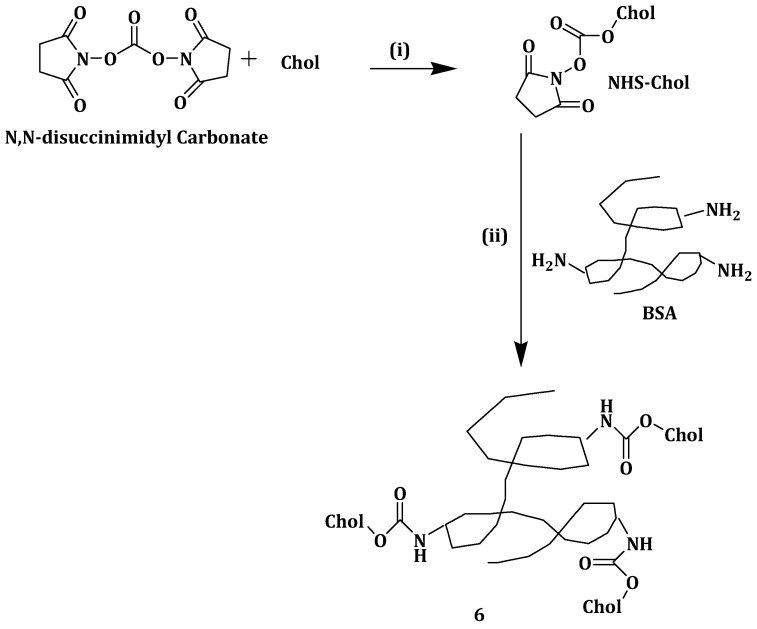
Synthesis of cholesteryl bovine serum albumin nanoparticles (**6**): Reagents and conditions: (i) THF, DSC, DMAP, 24 h, rt under argon; and (ii) THF, stir, 12 h.

Abraxane is a nanoparticle composite of paclitaxel (PTX) and human serum albumin (HSA), approved for cancer [46,47]. The poor colloidal stability of abraxane in the blood has been found not to improve PTX serum half-life [48,49]. To circumvent the aforementioned problem, Battogtokh et al. synthesized cholesteryl bovine serum albumin (Chol-BSA) (Scheme 3) nanoparticles (**6**) as PTX carriers. When loaded with PTX, nanoparticles **6** showed a 94.8% drug loading efficiency and 37.9% drug loading capacity making the formulation a potential drug carrier than its closest competitors [50]. The nanoparticles had an optimum size of 150 nm and a negative charge surface making them stable in aqueous medium [51]. The release of PTX was two-fold slower from PTX-loaded nanoparticle **6** when compared to PTX-BSA over a period of 72h at 37 °C, showing the sustained drug release from nanoparticles **6**. When incubated with B16F10 cells, there was 2- and 0.5-fold increase in PTX uptake from the PTX-loaded nanoparticles **6** when compared to PTX-Cre/EtOH and PTX–BSA, respectively. After incubating PTX-loaded nanoparticles **6** with MCF-7 cells for 1 h, the uptake of the nanoparticles was reported to be 3.5- and 1.4-fold higher when compared to PTX-Cre/EtOH and PTX–BSA, respectively. These findings revealed the capability of the PTX-loaded nanoparticles **6** in delivering more drugs to the cells [50].

Cytotoxicity studies revealed that B16F10 and MCF-7 cells treated with 10 and 100 nM PTX-loaded nanoparticles **6** for 48 h exhibited lower cell viability when compared to the same cell lines that were treated with PTX-Cre/EtOH and PTX–BSA. No cytotoxic effect was displayed by nanoparticles **6**. In vivo antitumor studies showed that the tumor volume in mouse model was smaller than that of mice administered with saline and other groups. Tumor volume of PTX-loaded nanoparticles **6** was reported to be 46.94% at day 8 after administration, compared to 78.23% and 77.35% of PTX-Cre/EtOH and PTX–BSA, respectively. The results indicated that PTX anti-tumor effect can be enhanced using nanoparticles **6** as a nanocarrier [50].

Tamoxifen (TMX) is a drug used to treat estrogen receptor and progesterone receptor breast cancer [52]. To improve its cell penetration, entrapment and pharmacokinetics, Mazumdar et al. developed mPEG-b-(CB-{g-chol}-co-LA), a self-assembling cholesterol grafted lipopolymer, synthesized from poly(ethyleneglycol)-block-2-methyl-2 carboxylpropylenecarboxylic acid-co-poly (L-lactide) [mPEG-b-(CB-{g-COOH}-co-LA)] copolymer followed by the incorporation of cholesterol through carbodiimide coupling. TMX release studies were done at pH 7.4 and 5.6, with the latter representing the acidic tumor medium. After 96 h at pH 7.4, 72% of TMX was released from [mPEG-b-(CB-{g-COOH}-co-LA)] while 55% was released from methoxy–poly (ethylene glycol)-poly(D,L-Lactide) (mPEG-PLA) (used for comparison). After 96 h of incubation at pH 5.6, about 97% of TMX was released from both nanoparticles [53].

The TMX loaded mPEG-b-(CB-{g-chol}-coLA) lipopolymeric nanoparticles displayed higher cellular uptake efficiency and improved IC_50_ values of 22.2 μM in the breast cancer cell lines in 4T1 and 18.8 μM in MCF-7 compared to IC_50_ values of 27.6 μM and 23.5 μM, respectively, for the free TMX. The results also showed that at the above IC_50_ values, TMX loaded lipopolymeric nanoparticles induced cell death and arrested cell cycle at G0/G1 phase at the same rate as the free TMX. The pharmacokinetics results showed approximately 2.5- and 2.7-fold increase in the half-life and mean residence time, respectively, of TMX after incorporating it into lipopolymeric nanoparticles. The findings indicate that mPEG-b-(CB-{gchol}-co-LA) lipopolymeric nanoparticles are useful for the internalization and enhanced residence time in breast cancer cell lines with reduced drug dose and side effects [53].

**Scheme 4:**
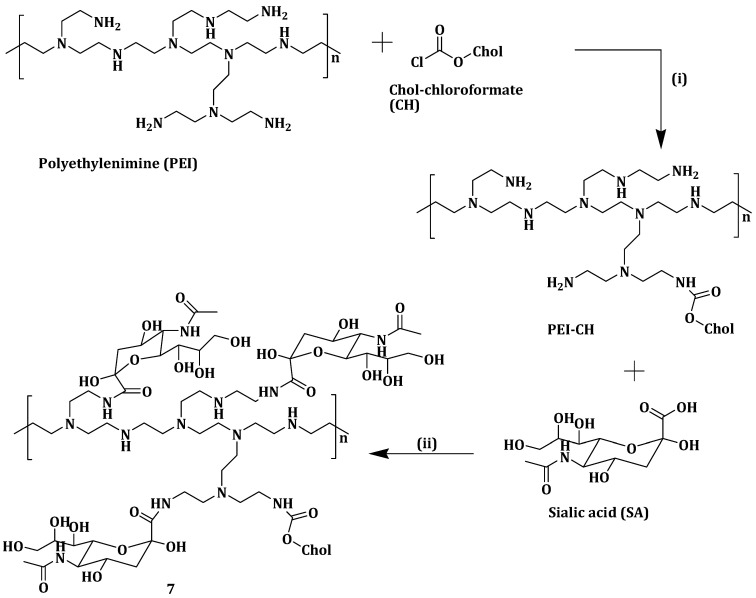
Synthesis of SA-PEI-CH conjugates (**7**). Reagents and conditions: (i) TEA, DCM; and (ii) EDC/NHS, Formamide, stir, 0.5 h, stir, 12 h under nitrogen.

Zheng et al. prepared sialic acid-polyethyleneimine-cholesterol modified liposomes loaded with doxorubicin (DOX-SPCL) (**7**) (Scheme 4) to enhance sarcoma chemotherapy. DOX-loaded liposomes **7** selectively bind to tumor associated macrophages (TAMs) when compared to the conventional liposomal doxorubicin (DOX-CL) and PEGylated liposomal doxorubicin (DOX-PL). DOX-loaded liposomes **7** did not display precipitation or aggregation which is attributed to its high zeta potential (−4.5 mV). Cellular uptake and intracellular trafficking studies showed that DOX-loaded liposomes **7** being trapped in RAW264.7 cells lysosomes up to 1 h [54].

Modification of the liposomes with sialic acid-polyethyleneimine-cholesterol enhanced their selective uptake through the binding of sialic acid and above all, the trafficking of DOX-loaded liposomes **7** into the cytosol is very critical for killing TAMs. The cytotoxicity of the liposomes was significant [55]. However, it is important to indicate that polyethyleneimine display cellular toxicity due to its structure, charge, etc. Compared with other organs, DOX-loaded liposomes **7** accumulated in the tumors and promoted exhaustion of TAMs. DOX-loaded liposomes **7** reduced tumor volume by 92.7% in vivo [54].

Cyclodextrins (CDs) are oligosaccharides whose free hydroxyl groups can be used for crosslinking either between CD molecules or with other molecules. CD nanosponges (NSPs) have been reported to improve absorption, solubility and control drug release [56]. Drugs can be incorporated within the CD NSP structure, but their applications suffer from their inability to interact with cell membranes and also their non-binding nature. Cholesterol hydrogen succinate (CHS) is known for its protein interaction and cellular binding properties [57]. Singh et al. functionalized β-cyclodextrin nanosponge (β-CD-NSP) surface with CHS in an attempt to enhance the cellular binding of CD-NSP. DOX was used as the model drug for cellular uptake and absorption capacity studies [57].

β-CD-NSP-CHS exhibited a 5.8% higher drug adsorption capacity when compared to β-CD-NSP. The enhanced drug adsorption is attributed to the hydrophobic nature of the surface. The DOX release profiles at pH 6.8 and 1.2 of β-CD-NSP-DOX and β-CD-NSP-CHS-DOX were the same, suggesting that CHS grafting did not change the release pattern. The drug release from both nanosponges at pH 1.2 was fast with over 82% of the drug released within 15 min when compared to phosphate buffer at pH 6.8 in which less than 30% of the drug diffused from the NSP [57].

Cytotoxicity studies revealed that β-CD-NSP-CHS exerted no cytotoxic effects in HeLa cells even at a high sample concentration of 100 µg/mL. The surface-modified β-CD-NSP was biocompatible and safe in drug delivery. Laser scanning confocal microscopic images showed high DOX accumulation in HeLa cells incubated with DOX-loaded β-CD-NSP-CHS samples when compared to HeLa cells treated with free DOX. This finding indicate that CHS played an important role in enhancing the drug penetration and cell internalization [58]. The surface modified CD-NSP is useful in the delivering of small low water-soluble drugs thereby improving both their solubility and bioavailability [57].

Mallick et al. prepared liposomes from cholesterol-phenylalanine-arginine-phenylalanine-lysine (Chol-FRFK) sequence and 1,2-dioleoyl-*sn*-glycero-3-phoshphoethanolamine (DOPE) was used to formulate (Chol-FRFK/D liposomes) for delivery of an anticancer drug, Antimycin A (AMA), to the mitochondria. The Chol-FRFK/D liposomes had a higher cellular uptake and mitochondrial targeting compared to the two controls, DQAsomes and DOTAP/DOPE (D/D) liposomes. Treating A549 cells with Chol-FRFK/D liposomes showed an IC_50_ of 10 µM compared to an IC_50_ of 50 µM for free AMA [15]. Drug encapsulation in the Chol-FRFK/D liposomes was 100% since AMA could be accommodated both in the lipid bilayer membrane and the inside of the core. The Chol-FRFK/D-AMA formulation was very cytotoxic that it caused mitochondria-mediated apoptosis in A549 cells. The Chol-FRFK/D liposomes are a promising anticancer delivery system for anticancer therapy [15].

**Scheme 5:**
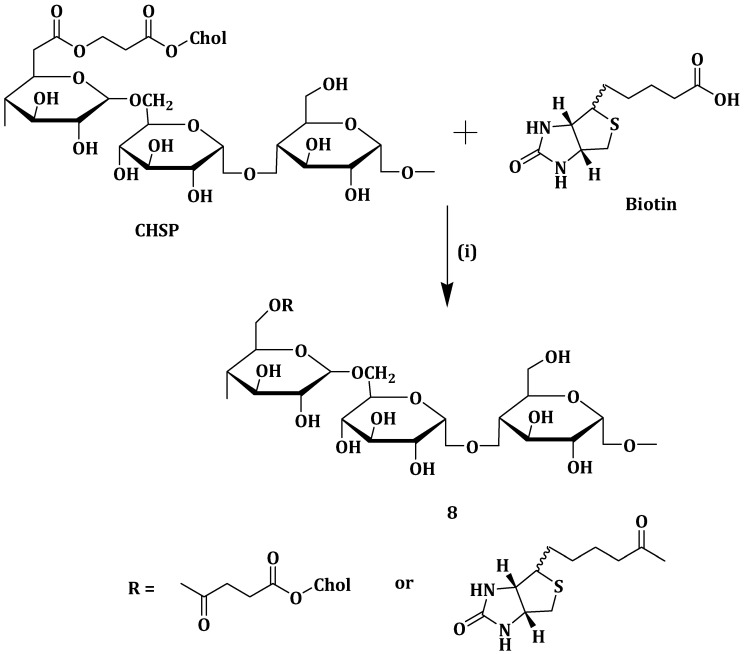
Synthesis of Biotin grafted- Cholesterol-modified pullulan co-polymer (**8**). Reagents and conditions: (i) EDC, DMAP, DMSO, 45 °C, 5d.

Cholesterol-modified pullulan nanoparticles (CHSP NPs) have been found to exhibit sustained drug release and excellent biocompatibility. Its major setback is that it does not actively target the tumor cells [59]. To deal with the above set-back, Yang et al. synthesized biotin grafted CHSP copolymer (Bio-CHSP) (Scheme 5) (**8**) for targeted drug delivery. Mitoxantrone (MTO) is an anticancer agent use in treating various carcinomas and it has side effects such as vomiting, nausea, cardiotoxicity and anemia. The synthesized nanoparticles **8** was used as a carrier for MTO to decrease its toxic effects, enhance therapeutic efficacy and for sustained drug release [60]. Nanoparticles **8** with different degree of substitution of biotin per hundred glucose units was synthesized (Bio-CHSP_-20.1_, Bio-CHSP_-29.2_ and Bio-CHSP_-38.9_) and they had critical aggregation concentration values of 0.0055, 0.0017 and 0.00015 mg/mL, respectively. The nanoparticles **8** self-aggregated in aqueous media because of the hydrophobic cholesterol and biotin. MTO release at pH 7.4 and 3.5 from MTO-loaded nanoparticles **8** was slightly pH-dependent. MTO dissolved more in lower pH thus inducing drug release, thus MTO release was faster in pH of 3.5 when compared to pH 7.4 [60].

Release studies showed that free MTO was released over 90% within 8 h. MTO was released rapidly in MTO-loaded nanoparticles **8** and then slowly after 12 h, suggesting sustained release ability of nanoparticles **8**. In vivo toxicity of nanoparticles **8** was studied in mice. Judged from their eating habits, pathological changes and body weight data, the mice remained healthy throughout the experiment. Histopathological studies of major organs showed that there was no difference between the control and those administered with nanoparticles **8**. This means that the nanoparticles did not induce acute toxicity after intravenous administration at a dose of 200 mg/kg. Together with the other in vitro results, nanoparticles **8** was biocompatible and safe for hydrophobic drug delivery [60].

**Scheme 6:**
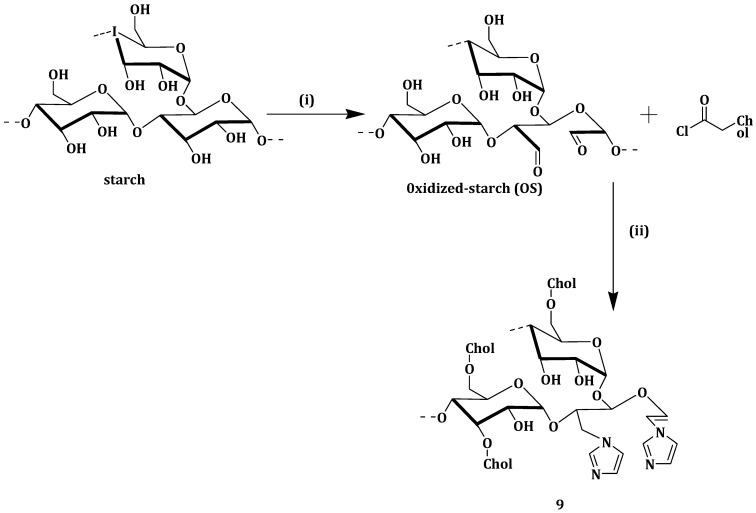
Synthetic route for Cho-Imi-OS (**9**). Reagents and conditions: (i) NaIO_4_, 3 h, 37 °C, dark; and (ii) Imidazole, 100 °C, 1 h, stir.

Xu et al. developed novel cholesterol/imidazole modified oxidized-starch (Cho-Imi-OS) (Scheme 6) (**9**) nanoparticles as a pH-sensitive and neutrally charged tumor-targeted drug delivery system, using curcumin as a model drug. The curcumin-loaded nanoparticles **9** drug loading and entrapment efficiencies were 4.16% and 17.84%, respectively. Curcumin was released more quickly at pH 5.5 (65% cumulative release) than at pH 7.4 (38% cumulative release) after 68 h. The faster cumulative release at pH 5.5 is very important since the pH in tumor cells endosomes is between 5.0 and 6.0, meaning most of the curcumin is released right at the tumor site for effective therapy [61].

The nanoparticles without a drug and curcumin-loaded nanoparticles **9** showed hemolysis ratios of 1% and 2%, respectively, which are all within the standard level of 5%. A549 cells effective uptake of curcumin-loaded nanoparticles **9**, when compared to free curcumin, was attributed to the nano-size of the curcumin-loaded nanoparticles **9**. Curcumin-loaded nanoparticles **9** exhibited an IC_50_ of 4.2 µg/mL, and the nanoparticles not loaded with drug maintained cell viability of more than 85% [61].

Wu et al. prepared liposomes with hydrogenated soybean phospholipids (HSPC), 1,2-distearoyl-*sn*-glycero-3-phosphoethanolamine-*N*-[methoxy(polyethylene glycol)-2000] (DSPE-PEG_2000_) and varying amounts of cholesterol (Table 1) and loaded them with doxorubicin. The rate of drug release decreased as the cholesterol content in the liposomes increased. Liposomal cellular uptake in BxPC-3 and HPaSteC cells decreased with increase in the liposomal cholesterol content. The authors reported a decrease in the membrane rigidity of the liposomes as the cholesterol content increased. The aforementioned findings suggest that when the liposomal membranes are less rigid, there is an increase in surface contact with the cells resulting in a prolonged cellular uptake [62].

Lip3 and Lip2 displayed moderate rigidity inhibiting tumor spheroid growth and exhibited high tumor penetration with excellent tumor growth inhibition in vivo. The results showed that moderate liposomal rigidity resulted in better diffusivity making cholesterol-tuned liposomes a promising system for delivery drugs to tumor cells [62].

Qui et al. prepared and patented a method for preparing liposome comprising of a phospholipid and cholesterol-based compound to deliver chloroquine (CQ) and doxorubicin (DOX). Liposomes were prepared from 500 mg phosphatidylcholine and 100 mg cholesterol and had an average size of 129 nm. These liposomes were loaded with 20 mg of CQ and DOX (1:1 *w*/*w*) and had a drug encapsulation of 96% and 97% and drug loading of 1.5% and 1.6% for CQ and DOX, respectively [63].

Cytotoxicity tests were performed using free drugs (CQ and DOX) as controls and no significant difference between free doxorubicin and drug-loaded liposomes was observed. Both displayed an IC50 = 0.4 µg/mL (Table 2) against human breast cancer cells (MCF-7). However, the drug-loaded liposomes were more potent on DOX-resistant MCF-7 cells (MCF-7/ADR) with IC_50_ = 3.3 µg/mL when compared to free DOX with IC_50_ = 13.7 µg/mL. Free CQ was less effective when compared to the drug-loaded liposomes on MCF-7 and MCF-7/ADR. The results followed a similar trend when the above formulations were used on human promyelocytic leukemia cells (HL60) and DOX-resistance human promyelocytic leukemia cell (HL60/ADR), as shown in Table 2. The results showed the potential ability of the DOX and CQ-loaded liposomes in overcoming mediated multidrug resistance [63].

### 3.3. Copolymers

Polymeric nanocarriers transport loaded drugs to the tumor cells [64]. They suffer from several barriers during transportation such as inability to diffuse through the cell membrane [65]. The biological pH stimulus can be used to facilitate drug release in acid-labile polymeric nanocarriers. Polymeric nanocarriers need to be constructed with strong bonds that can maintain an integrated structure [66,67].

**Scheme 7:**
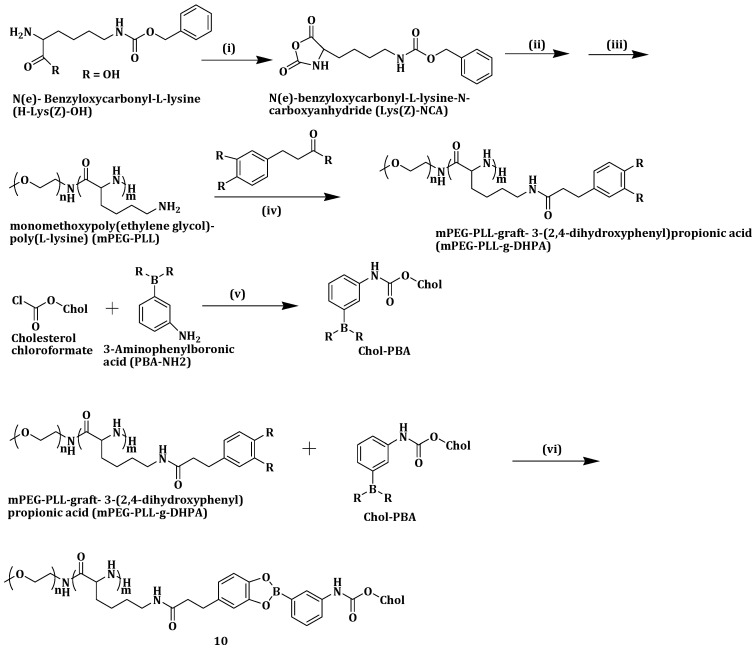
Synthetic route of MPDP (10) via boronate linkage. Reagents and conditions: (i) C_3_O_3_Cl_6_, THF, stir under N_2_ for 2 h at 50 °C; (ii) mPEG-NH_2_, DMF, stir under N_2_ for 72 h at 40 °C; (iii) CH_3_COOH, HBr, 1.5 h, stir; (iv) PyBOP, HOBT, DIEA, 24 h, stir, r.t.; (v) (CH_3_CH_2_)_2_O, N-Methylimidazole, 24 h, stir, r.t.; and (vi) THF/H_2_O, 48 h, stir.

Yang et al. constructed a pH-responsive polymeric drug carrier by reversibly attaching phenylboronic acid-modified cholesterol (Chol-PBA) to catechol-pending methoxypoly (ethylene glycol)-block-poly(L-lysine) (Scheme 7). The pH-dependent metastability of monomethoxypoly(ethylene glycol)-poly(L-lysine)-graft-3-(2,4-dihydroxyphenyl)propionic acid (mPEG-PLL-g-DHPA)/Chol-PBA (MPDP) (**10**) nanoassemblies was evaluated using Nile Red Dye (NRD) at a pH of 7.4 and 5.0 over a period of 24 h. No color changes were detected in the solution at pH 7.4. However, at pH 5.0, red deposits were observed with the solution color fading and ultimately becoming pale yellow. The co-polymer, **10** did not change size at pH 7.4 but increased sharply at pH 5.0 from 196.2 to 954.8 nm, indicating its structural instability in the acidic environment. The boronate bond hydrolyzed in acidic medium precipitating cholesterol and loaded NRD [68].

In vitro drug release studies were performed on **10** loaded with DOX. At a pH of 7.4, less than 40% of DOX was released within seven days, compared to 70% drug release at pH 5.0. Tumor HeLa cells viability in **10** remained very high above 90% at 500 µg/mL. Cellular uptake studies revealed that the internalization of the DOX-loaded **10** nanoassemblies into cells was via LDL receptor-mediated endocytosis [68].

**Figure 4:**
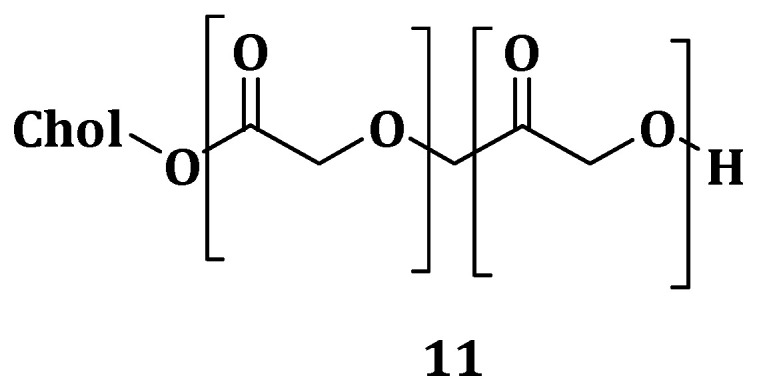
Chemical structure of PLGA-C (**11**).

Synthetic and natural polymers have been used for the intravenous delivery of anticancer agents to the tumor sites [69]. Poly(lactide-co-glycolide) (PLGA) is a biodegradable and biocompatible polymer for tumor-targeted drug delivery. Lee et al. developed poly(lactide-co-glycolide)-cholesterol based nanoparticles (PLGA-C NPs) (**11**) (Figure 4) for intravenous delivery of curcumin to the tumor and cell imaging. Curcumin-loaded nanoparticles **11** size was maintained in both PBS of pH 7.4 and serum (50% *v*/*v*), which is suitable for efficient drug delivery [70]. Curcumin release was performed at pH 5.5 (for endocytic compartments), 6.8 (for tumor environment) and 7.4 (for normal physiological conditions). The release of curcumin was in a pH-dependent manner with the amount of drug release after 144 h being 60.7%, 43.7% and 11.8% at pH 5.5, 6.8 and 7.4, respectively. The sustained and pH-dependent drug release mechanism of the drug-loaded nanoparticles influenced its drug tumor-targeting capability [70].

The cytotoxicity of the nanoparticles was evaluated in Hep-2 cells derived from human laryngeal carcinoma. Both PLGA NPs and the nanoparticles **11** showed no cytotoxic effects making them safe formulation for intravenous drug delivery. In vitro cellular uptake was assessed in Hep-2 cells by encapsulating Dil, a hydrophobic fluorescent dye, into the NPs. The nanoparticles **11** cellular accumulation efficiency was 1.6-fold higher when compared to the nanoparticles when incubated for 4 h. After 24 h incubation, the cellular distribution of the nanoparticles done by CLSM showed stronger fluorescence intensity in **11** when compared to PLGA NPs. In vivo tumor targeting of **11** was shown by Near-infrared fluorescence (NIRF) images of Hep-2 tumor-xenografted mouse model. The results revealed the usefulness of **11** in targeting tumor cells [70].

**Scheme 8:**
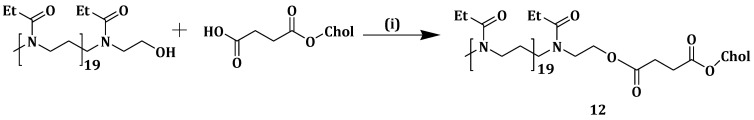
Synthetic route of PEtOz-CHEMS (**12**). Reagents and conditions: (i) DCC, DMAP, DCM, r.t, 10 h.

Incorporation of PEG into liposome surfaces is reported to increase drug bioavailability and the circulation time [71]. PEGylation of liposomes suffers from drawbacks such as loss of response in low pH environment and liposomal cellular uptake hindrance [72,73]. Poly(2-ethyl-2-oxazoline) (PEtOz), a long-chain macromolecule polymer, is biocompatible, flexible and low cost and has been reported as a suitable pH-sensitive drug delivery vehicle [74,75]. Similar to PEG, PEtOz increases liposome stability in vitro and in vivo, thus it can be used as a PEG substitute [76]. Xu et al. constructed pH-sensitive liposomes from poly (2-ethyl-2-oxazoline) cholesterol hemisuccinate (PEtOz-CHEMS) (Scheme 8) (**12**) and encapsulated DOX, an anticancer drug. DOX-loaded liposomes **12** displayed slow DOX release when compared to the conventional DOX liposomes (CL-DOX), CHEMS modified DOX liposomes (CH-DOX) at pH 7.4. DOX release increased rapidly as the pH decreased and more than 90% was released within 4 h at pH 5.4. This revealed the significant change in release kinetics at low pH and the stability of the formulation at normal physiological pH, after PEtOzylation of liposomes [77].

Cellular uptake studies after incubating A375 cells with the different liposomes showed that DOX-loaded compound **12** liposomes exhibited higher fluorescence intensity at pH 6.5 when compared to pH 7.4. PEtOzylation increased the cellular uptake of the liposomes with a maximum DOX accumulation, and, at pH 7.4, DOX-loaded liposomes **12** exhibited cellular uptake higher than that of CH-DOX, CL-DOX and PEG-DOX liposomes. CLSM analysis of A375 cells incubated with the different liposomes showed that PEtOz-DOX liposomes exhibited enhanced cellular uptake, and DOX release into the cytoplasm for PEtOzylated liposomes. A375 cells inhibition increased when incubated by the various liposomes at 37 °C under different pH. However, DOX-loaded liposomes **12** had higher cytotoxic effect at pH 6.4 when compared to pH 7.4 while CH-DOX, CL-DOX and PEG-DOX liposomes showed no difference in the inhibitions even at the different pH mediums. The results revealed the potential of PEtOz in modifying liposomes for drug delivery [77].

**Scheme 9:**
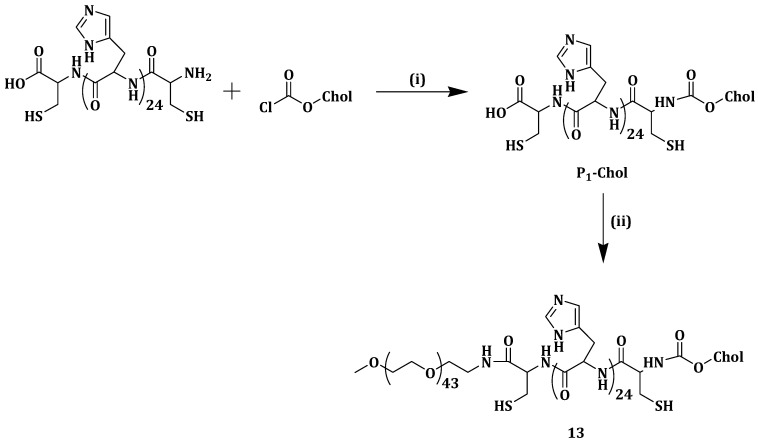
Synthetic route of mPEG-P_1_-Chol (**13**)**.** Reagents and conditions: (i) DMF, Triethyamine (TEA), rt, 72 h; and (ii) DMF, EDC-NHS, rt, 24 h.

The pH around and inside tumor cells is different from that of normal ones. The extracellular pH around cancer cells is reported to be weakly acidic around 6.5–7.0, while, inside tumor cells, the pH is between 5.0 and 6.5 [78]. Researchers have focused on developing pH-triggered drug delivery vehicles that are stable at normal physiological pH of 7.4 and can release drugs rapidly in the weakly acidic environment [79,80]. In their quest for a novel hydrophobic pH-sensitive drug carrier with lower toxicity and higher efficiency, Yang et al. designed and synthesized three triblock copolymers poly(ethylene glycol) methyl ether-b-peptide-g-cholesterol (mPEG-b-P-g-Chol) (Scheme 9) (**13a**–**c**), which self-assembled into micelles in an aqueous medium. The peptides functioned as the pH-sensitive part and the internal core comprising of cholesterol offered space for drug loading. DOX was loaded as the model drug [81]. The three polymers (mPEG-P1-Chol **13a**, mPEG-P2-Chol **13b** and mPEG-P3 Chol **13c**, which differed only in shape, being linear, y-shaped and fork-shaped, respectively) showed very low CMC values revealing that the micelles could remain intact even in extremely dilute volume of the body’s systemic circulation. The three DOX-loaded polymers exhibited larger particle sizes at a lower pH of 5.0 when compared to pH 7.4. The DOX loading in the three copolymers **13a**–**c** micelles was about 15.7%, 20.2% and 23.1% in weight, respectively, thus drug loading content and entrapment efficiency increased with polymer complexity [81].

In vitro drug release of the three polymers in solutions of pH 7.4 and 5.0 showed that DOX release was pH dependent. After 24 h, only about 35%, 30% and 26% of DOX was released from micelles **13a**–**c**, respectively. The three polymers exhibited DOX release of about 90%, 84% and 80%, respectively, in 148 h. The enhanced positive charge on micelles surface at pH 5.0 caused the histidine moieties to experience electrostatic repulsion increasing the swelling and drug release. At 400 mg/L polymer concentration, cell viability against NIH 3T3 cells was 97.7%, 95.5% and 95.4%, and 96.8%, 95.9% and 95.0% against HepG2 cells, for **13a**–**c**, respectively [81]. The DOX-loaded micelles exhibited lower cytotoxicity which increased slowly in lower concentrations suggesting a slow and time-controlled DOX release from the micelles. Although the free DOX-HCl showed the lowest cell viability, it was toxic to the tumor and the normal cells. The slight slowing down of the inhibition effect of DOX-loaded micelles **13** revealed their capability to protect the drug. The results showed the potential of **13** in delivering anticancer drugs [81].

**Scheme 10:**
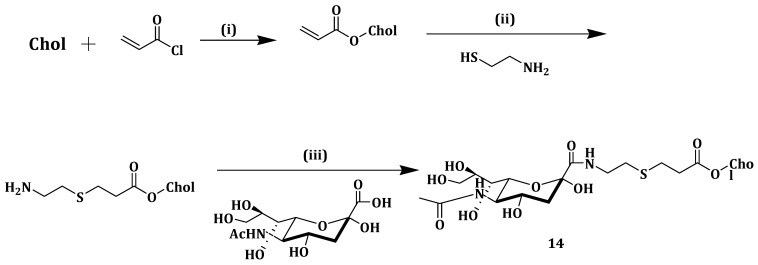
Synthesis of SA-Chol (**14**). Reagents and conditions: (i)TEA, DCM, 0 °C, 1 h, r.t.; and (iii) EDC, DMF, stir, 1 h, r.t.

Tumor cells are surrounded by different kinds of cells such as fibroblasts and inflammatory cells, mainly lymphocytes and macrophages [82]. The mass population around the tumor cells is termed tumor-associated macrophages (TAMs) and tends to promote tumor growth [83]. Siglec-1 is a member of the Sialic acid (SA) binding family that is overexpressed on TAMs. SA can be used as a ligand for the enhanced distribution of anticancer drugs to TAMs since studies have revealed that Siglecs function as endocytic receptors [84]. In trying to target TAMs and improve the tumor selectivity and specific toxicity of epirubicin (EPI), Zhou et al. synthesized a sialic acid-cholesterol conjugate (SA-Chol) (Scheme 10) (**14**) decorated onto EPI-loaded liposomes surface. Efficacy of **14** was studied in comparison with the sialic acid-octadecylamine conjugate (SA-ODA) [85].

When injected with 0.125 mmol/kg SA-ODA, mice death occurred within 1 min but no death was observed when the same dosage of **14** was administered to the mice used in the study. The aforementioned findings revealed the toxicity of SA-ODA when compared to **14**, making it a more favorable drug delivery system. In vitro release assay was done for the EPI-loaded **14** liposomes (EPI-SAL), EPI solution (EPI-S), conventional liposomal EPI (EPI-CL) and PEGylated liposomal EPI (EPI-PL). No significant EPI leaked from any of the liposomes and this suggested that incorporating **14** in the liposomes delayed EPI release to some extent. All the formulations proved to be stable for three months between 2 and 6 °C with no change in size and entrapment efficiency [85].

In vitro study on S180 and RAW264.7 cells using EPI-loaded liposomes **14** showed IC_50_ values of 1.41 and 0.47 µM (Table 3). However, EPI-loaded liposomes **14** had a better inhibitory ratio than EPI-CL especially with regard to RAW264.7 cells suggesting that SA moiety modification increased EPI cytotoxicity. In vitro cellular uptake studies revealed that **14** modification increased EPI cellular uptake in RAW264.7 cells when compared to EPI-CL. In RAW264.7 cells, SA solution inhibited EPI-loaded liposomes **14** cellular uptake due to the unavailability of SA receptor on the cells surface. Siglec-1 is overexpressed on the macrophage surface and it interacts with SA on the EPI-loaded compound **14** liposomes surface [85].

In vivo circulation studies showed that modifying the liposomes with **14** did not change its pharmacokinetic behavior. In vivo uptake of EPI formulations by TAMs showed that delivery of EPI to TAMs was greatly improved by EPI-loaded liposomes **14**. Compared with the other formulations, tumor-bearing mice injected with EPI-loaded liposomes **14** lived longer up to 60 days, denoting lower toxicity of EPI-loaded the liposomes. TAMs were identified by CD68 cells and fewer of them were noted when a group of mice was treated with **14**, indicating that it delivered EPI into TAMs thus destroying the macrophages. The results revealed that **14** has the potential to effectively deliver f EPI to TAMs [85].

**Figure 5:**
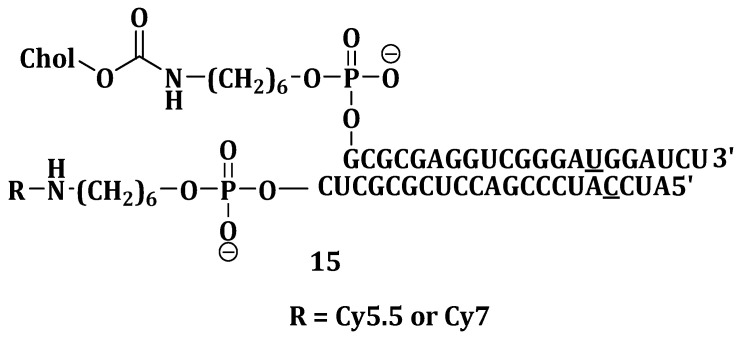
Structure of Chol-siMDR (**15**). 2’O-methyl nucleotides are underlined.

The potential of RNAi gene silencing technology in biomedical applications has been employed for a wide variety of diseases, especially genetic disorders and cancer [86]. Small interfering RNAs (siRNAs) can be chemically modified as a way of improving their therapeutic properties [8]. This makes them not to be easily degraded in the serum, and, thus, they are delivered to the target cells and tissues. The bioavailability and biodistribution of siRNA were discovered to be altered by substances such as antibodies α-tocopherol and cholesterol [87]. Chernikov et al. investigated gene-silencing activity and carrier-free biodistribution of cholesterol-conjugated nuclease-resistant anti-MDR1 siRNA (Chol-siRNA) (Figure 5) (**15**) in healthy and tumor-bearing mice. Intravenous administration of fluorescent **15** showed that it rapidly spread in the mouse bloodstream within 5 min. Other administration routes such as intramuscular, intrapertoneal and subcutaneous showed slow spreading of fluorescent **15** [88].

Attaching cholesterol to siRNA significantly reduced its accumulation in the kidneys but at the same time enhanced its accumulation in the liver. Reduced accumulation and slower excretion in the kidneys indicate the ability of siRNA to accumulate in the tumor. It was confirmed by CLSM that the tumor and liver cells had **15** in their cytoplasm [34]. P-glycoprotein levels were reduced by 60% in human KB-8-5 xenograft that overexpressed the MDR1 gene after five days of injection. The Pglycoprotein level in tumors was successfully reduced by cholesterol-siMDR regardless of how the **15** was administered. These results showed great promise in using compound **15** as a therapeutic agent for reversing drug resistance in tumors [88].

**Figure 6:**
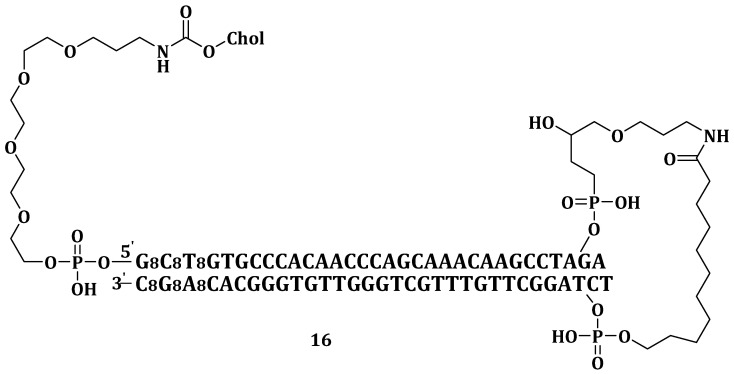
Structure of Chol-Dbait (**16**).

Sun et al. invented and patented an in vivo delivery system containing cholesterol and small DNA molecules (Dbait) (Chol-Dbait) (Figure 6) (**16**) and endosomolytic agents for nucleic acid. The components of the delivery system contribute to its capability to escape the endosome into the cytosol, impair the repair of DNA of damaged chromosomes thereby enhancing cancer treatment. The delivery system can be administered through a variety of routes but most preferably by intratumoral or subcutaneous injection. It required the assistance of a chloroquine as a fusogenic agent (which is administered separately) for its efficient release from the endosomes [89].

Irrespective of the route of administration, **16**, with or without chloroquine, was not toxic to the mice even up to doses of 800 mg/kg/injection. Lowest tumor growth was observed when SK28 xenografted human melanoma was first pre-treated with chloroquine followed by co-treatment with **16** and irradiated chloroquine. The formulation gave the best relative ratio of efficiency dose/toxicity dose of <0.037 when compared to 0.5 for the Dbait/PEI11K formulation. These results revealed **16**, a therapeutic agent suitable for clinical trials [89].

**Figure 7:**
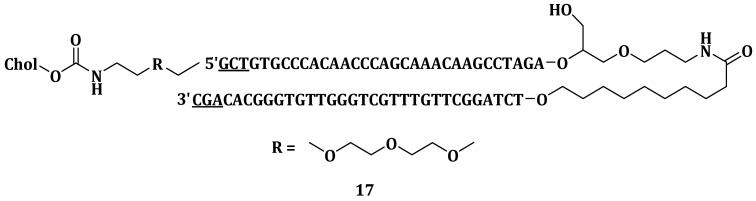
Structure of Chol-conjugated Dbait molecule (**17**).

Dutreix et al. invented therapeutic whereby hyperthemic treatment was combined with nucleic acid molecule mimicking double strand breaks (Dbait molecules **17**) for the treatment of solid tumors (Figure 7). The component contained Dbait, cholesterol (to facilitate endocytosis), chloroquine (as the endosomolytic agent) and an antitumoral agent for DNA damaging, most preferably selected from topoisomerases I or II inhibitors. Mice treated with the combination of **17** and hyperthermia exhibited an 80% survival chance compared to 30% survival of those treated which either of the treatment. The combination treatment also decreased tumor size since it caused the highest tumor destruction at degenerative and necrosis stages when compared to treatment with either hyperthermia or **17** alone [86].

## 4. Cholesterol-Based Carriers in Delivering Antimicrobial, Antiviral and Other Drugs

The continuous spread of microorganisms’ resistance to antibiotics is a major health problem that has led to researchers developing alternative classes of antibiotics. Antimicrobial peptides (AMPs) are essential polypeptides in the host defense, which play a vital role in the innate immune system. AMPs are promising agents for combating pathogens. The activity of AMPs can be possibly enhanced by improving their interaction with the membranes of the bacteria. Zhang et al. reported modified AMPs using steroids and fatty acids [90].

Antimicrobial peptides (AMPs) play important roles in the treatment of drug-resistant bacterial infections [91]. Their success has been attributed to their low drug resistance, multi-target effects and broad-spectrum antibacterial properties. Their use has been greatly limited because of the high chances of hemolysis after their systemic administration [92]. Zhang et al. synthesized Cholesterol-Modified Antimicrobial Peptide, DP7, with increased antimicrobial efficacy both in vitro and in vivo and reduced hemolytic activity. However, they exhibited vascular irritations and hemolysis under high intravenous dosage of DP7 [90]. To reduce the hemolytic capacity of DP7, it was conjugated with cholesterol (DP7-C). DP7-C exhibited a CMC of about 3.5 and 50 µg/mL in water and culture medium, respectively, suggesting its ability to self-assemble into micelles. DP7 was more toxic than DP7-C because all the mice injected intravenously with DP7 at a dosage 20 mg/kg died within 10 min while no fatalities were noted up to 144 h in the mice injected with DP7-C even at a dose of 80 mg/kg. The in vitro and in vivo studies further showed that the DP7-C micelles exhibited lower hemolytic activity when compared to DP7 [90].

The suppression of bacterial infection by DP7-C micelles was evaluated in zebrafish embryos infected with *Pseudomonas aeruginosa* strain 284 ATCC10145-GFP. The infected zebrafish embryos treated with DP7-C micelles showed that the micelles inhibited ATCC10145-GFP amplification in a time-dependent manner. A defensive immune reaction was stimulated in mice administered with DP7-C micelles in vivo. The results showed that DP7-C can significantly treat bacterial infection without adverse side effects [90].

**Scheme 11:**
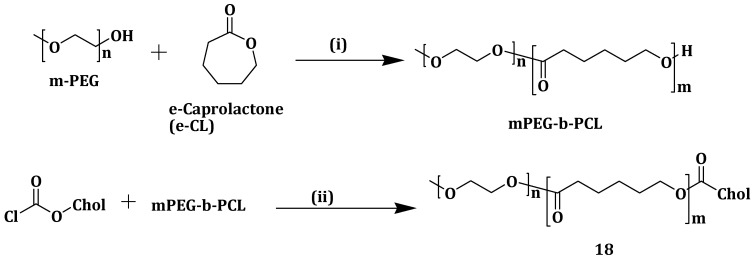
Synthesis of mPEG-b-PCL-Chol (**18**). Reagents and conditions: (i) Sn(EH)_2_/Toluene, 110 °C, 24 h; and (ii) N(CH_2_CH_3_)_3_, 40–60 °C, 48 h.

Villamil et al. conjugated a polymer of Methoxy-Polyethylene glycol and ε-caprolactone (mPEG-b-PCL) with cholesterol (mPEG-b-PCL-Chol) (Scheme 11) (**18**) in an attempt to attenuate the nephrotoxicity, increase loading capacity and aqueous solubility and encapsulation efficiency of Amphotericin B (AmB). The formulation, **18** had 60% drug encapsulation efficiency (%EE) compared with 28% and 42% of mPEG-b-PCL and mPEG-Chol, respectively (Table 4). The above values showed the role played by cholesterol in increasing the encapsulation of AmB in the polymeric micelles [93].

Micelles **18** drug release rate was slow, and this is attributed to the decreased critical micelle concentration, hydrophobic micelle core and more favorable AmB interactions. Micelles **18** not loaded with drug showed in vitro non-toxicity to fibroblast L929 cells with 80% cell survival at a dosage of 120 mg/L. AmB-loaded micelles **18** showed a noticeable increase in hemolysis. Nevertheless, it was 4 times less toxic than Fungizone, a standard fungicide [93].

Antifungal study was performed against *C. albicans SC5314* and AmB loaded micelles **18** was more effective when compared to mPEG-Chol due to the release profile of AmB (Table 4). In vivo results from *G. mellonella* infected with a *C. albicans SC5314* strain and then treated with the formulation and Fungizone showed that compound **18** loaded with AmB exhibited a survival of 80% after 7 days while all the non-treated larvae died [93]. The results showed that **18** did not only improved the encapsulation efficiency of AmB, but also increased its controlled release from the micelles, making it a promising drug delivery system for AmB.

Other diseases have been studied using cholesterol-based systems. Idiopathic pulmonary fibrosis (IPF) is a deadly disease with its patients having a mean survival of only 2–4 years. Signs and symptoms of IPF include injury of the alveolar epithelial cells, fibroblasts hyperplasia and accumulation of inflammatory cells. This results in extracellular matrix, including proteoglycans and fibrillar collagens to be deposited. Research on antimalarial drugs such as chloroquine (CQ) and hydroxychloroquine (HCQ) has shown that they exert anti-inflammatory activity by the down-regulating pro-inflammatory cytokines. HCQ effectively inhibits fibroblasts proliferation and suppresses metabolic activities in fibrotic skin diseases [94].

**Figure 8:**
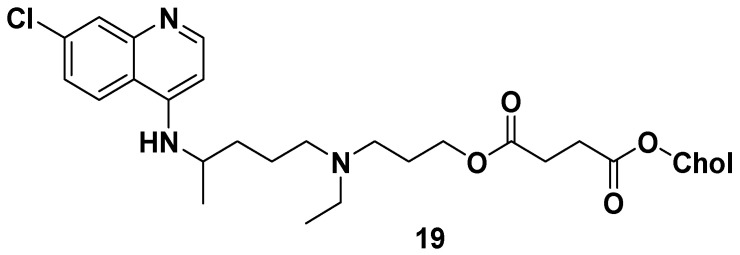
Cholesterol-modified HCQ (**19**).

Liu et al. synthesized cholesterol-modified hydroxychloroquine (Figure 8) (**19**). HCQ induces apoptosis in human dermal fibroblasts at concentrations of 1–10 μM. Compound **19** exhibited low toxicity against lung fibroblasts and inhibited cell proliferation at 10 μM, but it induced apoptosis at a higher concentration of 50 μM when compared to the unconjugated HCQ [94].

Compound **19** hindered early pulmonary inflammation. It also inhibited the CTGF/ERK signaling pathway which is effective against pulmonary fibrosis induced by bleomycin. The results revealed the capability of compound **19** to prevent bleomycin induced pulmonary fibrosis, revealing its anti-fibrotic activity useful for the treatment of inflammation caused by pulmonary fibrosis [94].

Viruses are intracellular parasites that depend on the host cells for life. A virus contains a nucleic acid genome, a protein capsid and a lipid envelope. The properties of the lipids (sphingolipids, glycerophospholipids and sterols) of an animal membrane affect the viral entry [95]. Hepatitis C virus (HCV) is a serious health condition due to its lack of an effective cure. It is the root cause of liver diseases such as cirrhosis, hepatocellular carcinoma and chronic hepatitis [96]. After the introduction of protease inhibitors to the interferon/ribavirin therapy, which showed improved virological activity, there was a need for better HCV antivirals because the inhibitors posed unwanted side effects and in only about 50% of the patients did the therapy led to a sustained antiviral response [97].

**Scheme 12:**
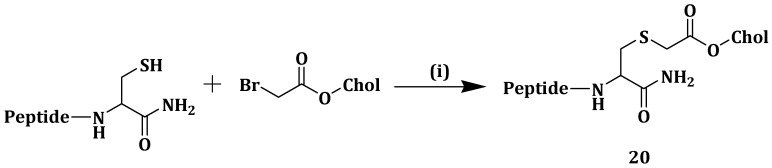
General synthetic route for cholesterol-derivatized peptides (**20**). Reagents and conditions: (i) NBoc-AmidoPEG4-acid, DIC, DMAP, DCM, 18 h, r.t, DMSO, THF, DIEA, r.t.

**Figure 9:**
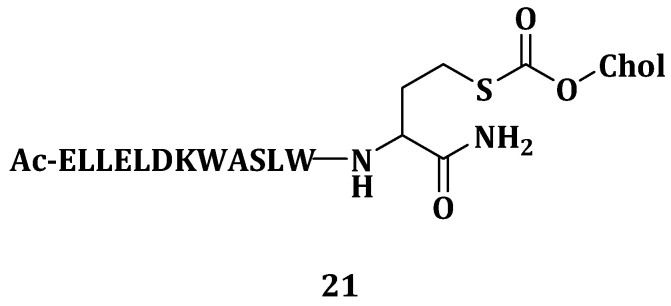
Structure of ELLELDKWASLW-Chol (**21**).

Pessi et al. prepared and patented a series of contiguous amino acids belonging to the HR2 domain of Type 1 viral fusogenic protein on an enveloped virus which they attached to the C-terminal of cholesterol (Scheme 12) (**20**) for viral fusion inhibition. The role played by the cholesterol moiety was exhibited by peptide ELLELDKWASLW, which on its own had an IC_50_ > 100 µM, but, when derivatized with the C-terminal of cholesterol (Figure 9) (**21**), its antiviral potency improved to IC_50_ = 5.85 µM. Based on Table 5, the antiviral potency of C34 increased 50-fold when conjugated to cholesterol (C34 and C34-Aceta have IC_50_ = 205 and 270 µM, respectively, compared to IC_50_ = 4 for C34-Chol) [98].

Chol-C34 showed 50-fold less antiviral potency compared to underivatized C34 because of the cholesterol that was linked to the N- instead of the C-terminal. C34-Chol was found to be approximately 100-fold more potent than enfuvieride, the marketed fusion inhibitor. C34-Chol was also more potent than all the controls against other HIV-1 isolates such as BAL, NL4-3, MN-1, etc. The compound **20** peptides are potent for viral fusion inhibition and also for efficacy of other therapeutic agents such as nucleoside inhibitors and limiters of HIV infection degree, which may be administered simultaneously or in alternating [98].

**Figure 10:**
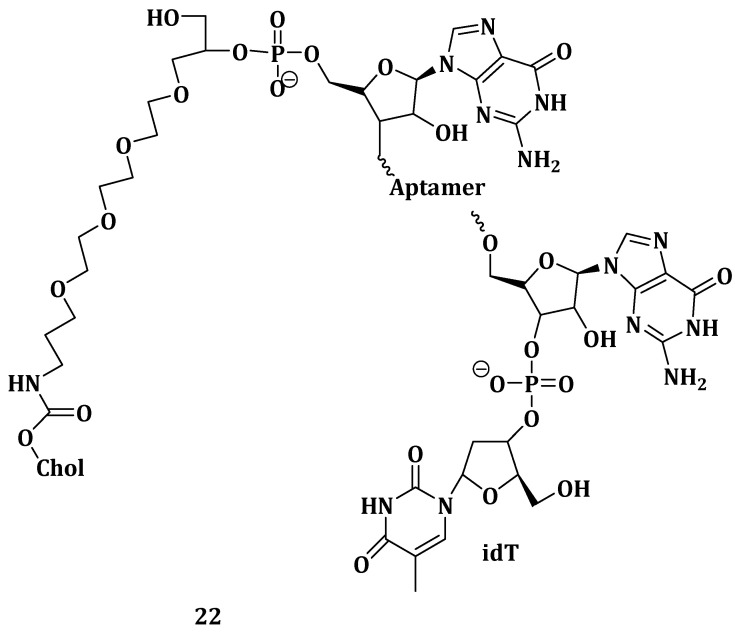
Structure of inverted d(T)-aptamer-Chol conjugate (**22**).

Lee et al. reported the efficient inhibition of HCV replication and its suppressed formation of HCV infectious virus [96] when the HCV nonstructural protein 5B was treated with a 29 nucleotide-long 2’-F pyrimidine modified RNA aptamer. The modified aptamer conjugated with cholesterol (Figure 10) (**22**) entered the cell efficiently and inhibited the replication of HCV RNA [86]. After systemic administration in mice, the cholesterol-aptamer exhibited high tolerance without abnormalities. The compound **22** also showed extended aptamer time in the plasma, increased dose proportionality and enhanced the exposure of aptamer to the body when compared to the free aptamer [96].

Pessi et al. developed cholesterol-conjugated stapled peptides as fusion inhibitors which were tested in vitro against a number of *Ebolavirus* species Ebola Zaire (EBOV), Sudan (SUDV), Chikungunya (CHIKV) and Marburg (MARV) viruses [99]. Cholesterol was used because of its ability to direct the inhibitor to lipid rafts, where fusion is known to occur, thereby increasing antiviral activity [100]. A PEG_4_ linker was used between cysteine and the cholesterol group. Eight peptides (EBOV-1- EBOV-8) were developed with different lengths depending on the number of residues, with the first five having PEG_4_ while the last three had PEG_12_. EBOV-4 inhibited all three tested viruses (EBOV, MARV and SUDV) effectively with EC_50_ values of 6.67, 6.21 and 3.03 µM, respectively. Since EBOV-4 was one of the shortest conjugates, it suggests that the efficacy of the conjugate was dependent on the length [99].

EBOV-5, EBOV-6, EBOV-7 and EBOV-8 were studied for their ability to inhibit EBOV and MARV GP-pseudotyped retrovirus cellular entry and their antiviral activity in rodent cells infected with EBOV. EBOV-7 was the most potent inhibitor in vitro, with EC_50_ values of 0.60 and 0.50 µM in vitro and in vivo respectively. Mice administered with EBOV-7 subcutaneously had a 100% survival while the other three peptides ranged between 10% and 40%. The results suggested that EBOV-7 could protect the mice from being infected by the lethal EBOV, giving a promising lead in finding an effective therapeutic conjugate against EBOV, which is still claiming lives in Western Africa up to date [99].

**Figure 11:**
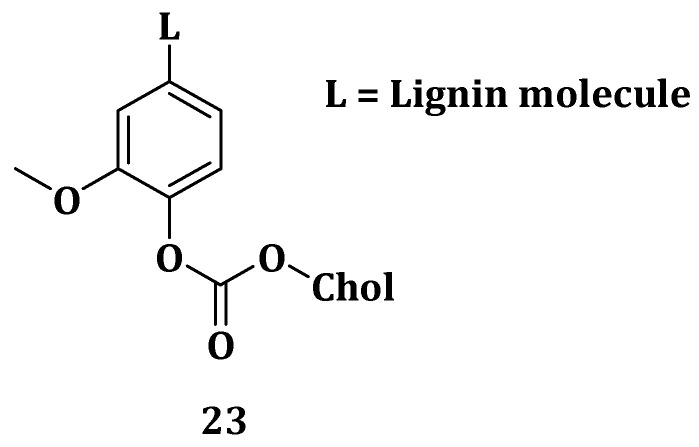
Structure of ASKL-Chol (**23**).

Pourmoazzen et al. prepared lignin-cholesterol nanoparticles with low molecular weight which were soluble in acetone (ASKL-Chol) (Figure 11) (**23**), as a drug delivery system. The nanoparticles were loaded with folic acid. When tested at pH media of 5.5, 7.4 and 8.0, folic acid was released more quickly at pH 8.0 with a sharp burst within the first 8 h then a sustained release for 40 h. Not all the folic acid was released from the nanoparticles possibly due to drug/carrier interactions [101]. It is biocompatible and a promising system for the delivery of hydrophobic drugs [101].

Jenkins et al. prepared and patented different types of self-responsive lipid vesicles made up of varying percentages of 1,2-dipalmitoyl-sn-glycero-3-phosphocho line (DPPC), 1,2-dipalmitoyl-sn-glycero-3-phospho ethanolamine (DPPE), 10, 12-tricosadiynoic acid (TCDA) and cholesterol (Table 6) for bacterial infection detection and wound dressing. Encapsulating carboxyfluorescein as the fluorescent molecule showed selective detection between pathogenic strains of *S. aureus* MSSA 476 and *P. aeruginosa* PAO1 and non-pathogenic bacteria *E. coli* DH5α. The vesicle further encapsulated an active therapeutic agent such as an antibiotic, bacteriophage or an antimicrobial [102].

The selective responses were shown by the different percentages of fluorescent responses exhibited by the different vesicle types to both PAO1 and MSSA476. For example, vesicle type 1 had a fluorescence response of about 50% to PAO1 and 80% to MSSA476 while vesicle type 2 showed 65% response to PAO1 and less than 10% for MSSA476. This was attributed to the different rates of lipids loss from the cell membranes of the bacteria. The different vesicle compositions affected membrane fluidity, exotoxins binding and cell membrane hemolysis, which all affected the sensitivity of the vesicles to the bacterial strains [102]. The vesicles were self-responsive, discriminatively sensing and selective, thus are potential cholesterol-based wound dressings and active drug delivery systems.

De Oliveira et al. synthesized cholesterol-functionalized poly (D, L-lactide-co-glycolide) (PLGA) nanoparticles to explore the antitumor, antioxidant and anti-inflammatory effects of loaded carvedilol (CVDL). CVDL-free nanoparticles (NP) and nanoparticles with different CVDL:PLGA ratios were prepared. Nanoparticles with a weight ratio of 1:10 for CVDL:PLGA had the highest drug loading efficiency of 95.06% and these were functionalized with cholesterol to give NP CVDL Chol. All the three nanoparticles (NP, NP CVDL and NP CVDL Chol) were spherical with smooth and uniform surfaces. In vitro anti-colorectal cancer studies showed that NP CVDL Chol was the most effective and it decreased CT-26 cell proliferation rate at a lower dose of 0.78 µg/mL when compared with CVDL which showed the same proliferation rate with 3.12 µg/mL dosage [103].

However, 0.35 µg/mL CVDL failed to induce cell death, same dosage of NP CVDL and NP CVDL Chol induced cell death, suggesting that the use of nanoparticles can use less amount of CVDL, thus preventing side effects associated with high doses. All the tested treatments showed their ability to prevent oxidative stress since malonyldialdehyde (a product of lipid peroxidation) levels remained low throughout the experiment. In vivo results showed that NP CVDL Chol (0.05 and 0.1 mg/kg doses) decreased leukocyte migration and increased glutathione levels more efficiently than free CVDL and NP CVDL. All the combined results showed the potency of cholesterol-functionalize PLGA nanoparticles in decreasing CVDL dosage without affecting its antitumor, antioxidant and anti-inflammatory effects [103].

## 5. Cholesterol-Based Compounds in Transdermal Drug Delivery

The healing of cutaneous wounds has four stages which are hemostasis, inflammation, proliferation and maturation [104]. Angiogenesis, which is the formation of new blood vessels, is an essential process during wound healing [105]. Tissue damage and delayed wound healing is attributed to an overproduction of reactive oxygen species (ROS) during the inflammatory phase. The body’s defense system releases endogenous antioxidant that helps against overexpression of ROS. A number of exogenous antioxidants such as phenolic compounds, ascorbic acid and vitamin E also speed up wound healing processes [104]. An ideal wound dressing needs to keep the wound moist, minimize infection, reduce pain and increase the rate of healing [106]. Wound dressings currently in use include nanofibers [107], peptide nanogels [106], niosomes [5], etc.

Niosomes are lamellar vesicles which are able to form colloids and they are prepared from mixing non-ionic surfactants and cholesterol [108]. The cholesterol used for the preparation of niosomes is useful for the maintenance of the rigidity and shape while the surfactant mainly provides a hydrophilic core for the localization of the hydrophilic drugs [24]. Niosomes are used as drug delivery system for hydrophilic and hydrophobic drugs. They are known to prolong drug half-life in blood circulation and also protect the encapsulated drug from environmental degradation [9]. Their flexibility allows them to penetrate into the deep skin layers hence they can be used for transdermal drug delivery [5,103]. This section reviews cholesterol-based compounds, such as niosomes and nanohydrogels, in transdermal drug delivery.

Imkan et al. synthesized biocompatible triazole-based niosomes (TBNs) and investigated their drug delivery capabilities loading Amphotericin B (AB), a broad-spectrum antifungal. The drug-loaded niosomes had a mean size of 279.9 nm, polydispersity index of 0.29 and zeta potential of −17.2 mV [109]. All three features played an important role in drug loading, stability and distribution of the entrapped drug [9]. The presence of cholesterol in the TBNs enhanced their high drug entrapment efficiency of 88.76%. In vitro drug release at pH 7.4 and 1.2 were found to be 70.90% and 61.97%, respectively, after 6 h [109]. When stored for up to 30 days, the AB-loaded TBNs retained 82.08% of the drug with a zeta potential of −16.5 mV. The AB-loaded TBNs were able to maintain an 84.22% drug content when subjected to simulated gastric fluids showing their ability to withstand the harsh gastric environment. The synthesized TBNs showed hemolysis of 20.12% when tested at 1000 µg/mL compared to hemolysis of 25.28% for Tween 80 at the same concentration. When subjected to NIH 3T3 cell line, the TBNs exhibited 60.78% cell viability compared to 53.00% of Tween 80. In vivo toxicity studies showed that the mice survived at a dose of 2000 mg/kg body weight with no side effects. In vivo pharmacokinetic studies also showed that the TBNs released AB in a slow and sustained manner with increased drug absorption via the gastrointestinal membrane. The results showed how TBNs prepared from cholesterol can increase AB efficacy and oral bioavailability [109].

To enhance patient compliance and reduce the dosing frequency of the anti-inflammatory drug aceclofenac, Fathalla et al. prepared noisome containing aceclofenac and evaluated in vitro and in vivo their gel performance and skin permeation. Different niosomes were prepared from non-ionic surfactant such as Span 20, 60 and 80 and the niosomes were incorporated into sodium alginate (7% *w*/*w*), hydroxyl propyl methylcellulose (HPMC) (3% *w*/*w*), pluronic F127 (20% *w*/*w*), sodium carboxyl methyl cellulose (3% *w*/*w*) and carbopol 924 (1% *w*/*w*). Niosomes of Span 60:cholesterol of ratio 0.5:0.5 had the highest entrapment efficiency, as high as 95.6% when encapsulated with 15 mg of the drug. Increasing cholesterol content generally increased noisome size with the highest being 22.39 µm [110]. In vitro drug release studies showed that the release pattern of aceclofenac ranked as: HPMC > sodium alginate > sodium CMC > carbopol 934 > pluronic F-127 gels. In vitro skin permeation using HPMC (3% *w*/*w*) niosomal gel showed high drug permeation through the hairless rat skin. The niosomal gels made of Span 20 and Span 60 showed 3.7–2.2 times higher flux values of the non-niosomal gels. Anti-inflammatory activity of aceclofenac from the HPMC (3% *w*/*w*) niosomal gels prepared from Span 20 on three male albino rats showed 85.89% paw edema inhibition compared to 37.16% for non-niosomal gel formulations after 6 h. HPMC niosomal gel formulation with Span 20:cholesterol of 0.5:0.5 exhibited sustained aceclofenac delivery [110].

Manconi prepared baicalin-embedded gellan-cholesterol nanohydrogels for wound healing. Baicalin sonicated nanohydrogel (BSN) and baicalin autoclaved nanohydrogel (BAN) were compared against empty sonicated nanohydrogels (ESN) and empty autoclaved nanohydrogel (EAN) and characterized, as displayed on Table 7. The negative zeta potentials confirmed the stability of the nanohydrogels upon storage. In vitro skin delivery of baicalin showed similar drug accumulation in stratum corneum and epidermis. Treatment of 3T3 cells with hydrogen peroxide and baicalin showed that baicalin counteracted the oxidative effect of hydrogen peroxide with a 95% cell viability, irrespective of the dosage administered (20, 10, 1 and 0.1 µg/mL). These results showed the excellent antioxidant capacity of baicalin [111].

In vivo wound healing activity of baicalin was studied by injuring the back skin of mice using topically applied 12-O-tetradecanoylphorbol 13-acetate (TPA) followed by treating the lesions with baicalin. The results showed that baicalin-loaded formulation healed the TPA lesions and inhibited inflammation markers more efficiently than baicalin in PBS (10 mg/mL) and even dexamethasone, a potent anti-inflammatory drug. BAN inhibited two inflammation markers, edema and myeloperoxidase (MPO), by 78% and 44%, respectively, while betamethasone cream showed no effective inhibition. BAN also showed 100% cytokine inhibition. These results showed the potential use of the autoclaved nanohydrogel in topical wound treatment and other skin diseases caused by oxidative stress and inflammation [111].

Farmoudeh et al. prepared methylene blue (MB)-loaded niosomes using Span 60, Tween 60 and cholesterol in different ratios. The MB-loaded niosomes had a maximum size of 613.76 nm, a maximum entrapment efficiency of 75% and negative zeta potential values. Increasing the cholesterol to surfactant ratio decreased the vesicle size. The authors used equations to prepare an optimized MB-loaded niosomal formulation with 147.8 nm vesicle size, −18.0 mV zeta potential and 63.27% entrapment efficiency. The optimized niosomal dispersion exhibited a controlled MB release rate with 26.897% of the drug released in the first hour compared to 45.064% of the free drug. In vivo results from the wounded male Wistar rats administered the MB (niosomal gel of free) transdermally showed significant wound closure when compared to those treated with placebo gel that was still open after 14 days [104]. The niosomal-gel treated wounds had thicker, organized collagen fibers with more fibroblasts and less inflammatory infiltrate compared to thin granulation tissue, disorganized collagen fibers and high number of inflammatory cells exhibited in free MB-treated and control rats. Niosomal gel-treated rats had significantly decreased malondialdehyde (MDA) and elevated Superoxide dismutase (SOD) levels only three days after surgery, but it took seven days for negative controls to reach the similar results [104]. Since SOD is an endogenous antioxidant and MDA is an end product of lipid peroxidation, the increase of the former and decrease of the latter showed the ability of niosomal gel in accelerating the rate of mitochondrial cytochrome c reduction thereby reducing the production free radicals in the mitochondria [112].

Aziz et al. prepared niosomes for efficient transdermal delivery of diacerein (DCN) and avoiding gastrointestinal problems associated with its oral administration. The niosomes were prepared from cholesterol and Span 40, Span 60, Span 80, Tween 20 or Tween 80 in varying ratios, with only Span 40 and Span 60 giving milky vesicular dispersion upon hydration. The optimal DCN-loaded niosomes had an entrapment efficiency of 95.63%, particle size of 436.65 nm, polydispersity index of 0.47 and −38.80 mV zeta potential. The optimal DCN-loaded niosomes (optimized using central composite design) were prepared in hydration medium with no salt and with a lipid:surfactant ratio of 150 mg:5 parts. The optimal niosomes composition was regulated using edge activators to give elastic vesicles for comparing with optimal niosomes [113]. The optimal niosomes enhanced the skin permeation of DCN ex vivo with up to four times better than the drug suspension. In vivo skin deposition of DCN expressed by AUC_0−10_ was four folds for elastic vesicles (65.8 µg h/cm^2^) and two folds for the optimized niosomes (32.7 µg h/cm^2^) compared to 16.7 µg h/cm^2^ for the drug suspension. The elastic vesicle was more effective than the optimal niosomes and improved drug retention and deposition within the skin [114]. The optimized niosomes proved their ability to transdermally deliver DCN, avoiding problems associated with oral administration [113].

Shirsand et al. prepared niosomal gel formulations for the delivery of ketoconazole (KTZ), to improve its fungal skin treatment efficiently. Niosomes consisting of different ratios of Span 40, Span 60, Tween 60 and cholesterol were prepared (Table 8). The vesicles had a spherical shape and a smooth surface. Niosomes with the highest cholesterol content (TNS3, TNS6 and TNS9) displayed small particle size (Table 9). Increasing cholesterol content, increased the entrapment efficiency of KTZ. The entrapment efficiency (EE) increase was attributed to the ability of cholesterol to close spaces between the bilayer membranes preventing drug leakage [115].

In vitro studies showed the drug release was in the range of 46.63–72.37% with the drug being released by both diffusion and erosion. Though TNS1 had the highest cumulative drug release after 24 h, TNS4 was found to be the best when combining vesicular size (5.94 µm), EE (69.39%) and drug release (66.06%). TNS4 was incorporated into a carbopol gel for further studies. TNS4 has a pH of 5.56, a drug content of 96.425 and 8370 cps viscosity. TNS4, plain gel and a marketed ointment had in vitro drug release studies of 36.18%, 73.21% and 82.06%, respectively, after 12 h showing the sustained drug release for TNS4. In vitro antifungal activity studies revealed that TNS4 had the highest inhibition zone of 25.3 mm compared to 19.6 and 23.3 mm for the ketoconazole plain gel and marketed ointment. Thus, the antifungal activity of KTZ against skin infections can be enhanced by loading it into niosomal gel formulations [115].

**Figure 12:**
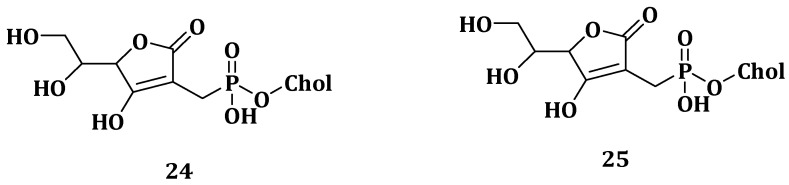
Chemical structures of compounds **24** and **25**.

Ptchelintsev et al. patented the novel uses of 3’-(L-ascorbyl-2-o-phosphoryl)-cholesterol (**24**) and 3’-(L-ascorbyl-3-o-phosphoryl)-cholesterol (APC compounds) (Figure 12) (**25**), which include reducing the synthesis of epidermal abnormal elastin and achieving the antioxidant activity of the topical compositions. Cultured fibroblasts in a medium containing compound **24** was discovered to have a decreased elastin promoter activity in a dose-dependent manner. Compound **24** also inhibited elastin promoter activity for a longer period of time than did ascorbic acid and significantly increased the synthesis of triglyceride in the cultured human keratinocytes [116].

Compound **24** showed effective hydroxyl radicals scavenging activity and inhibited superoxide formation by 50%. It exhibited the ability to produce new human skin fibroblasts. The results showed the capability of APC compounds in reducing epidermal synthesis of abnormal elastin and their antioxidant activities [116].

Lopinavir is a highly recognized protease inhibitor used for treating AIDS. Its oral administration is severely limited because of its moderately high molecular weight and poor aqueous solubility [117]. Patel et al. prepared a niosomal gel for the transdermal delivery of lopinavir in order to improve its bioavailability. Different formulations were prepared with different compositions of Span 40 and cholesterol (Table 10) [118]. Entrapment efficiency (EE) of lopinavir improved with an increase in cholesterol content probably due to better lipid bilayer stability and lipophilic behavior [119,120]. Generally, the vesicular size decreased when there was a decrease in the content of Span 40 and an increase in the content of cholesterol [118].

Etodolac (ETD) is a non-steroidal anti-inflammatory drug whose application suffers from poor solubility, short half-life and undergoes quick s first pass metabolism when administered orally [121,122]. Asthana et al. investigated the potential of loading ETD into a topical niosomal gel for transdermal administration. Niosomal formulations with different ratios of Span 60 and cholesterol were prepared and dicetyl phosphate was added to improve the stability of the formulations over time. As cholesterol content increased, the size of the niosomes increased. Formulation N_2_ with a drug:cholesterol:Span 60 ratio of 1:1:1 had the highest drug entrapment efficiency of 96% (Table 11). The decrease in the drug entrapment efficiency as the amount of cholesterol increased above the ratio of 1 suggests that a competition of cholesterol and the drug for packing space in the bilayer [122].

The particle sizes of the niosomal formulations were in the range of 2–4 µm. The particle sizes increased with increase in the content of cholesterol. When the content of cholesterol is low, there is a close packing of cholesterol and nonionic surfactant with increased curvature and reduced size. When the content of cholesterol is increased, the content of surfactants decreased and the hydrophobicity of the bilayer membrane increased, thereby increasing the vesicles radius and establishing a thermodynamic stable form. Furthermore, the bilayer membrane displayed a rigid structure due to cholesterol content, which results in reduced size from sonication and a bigger vesicles size [122].

In vitro studies performed in phosphate buffer solution at pH 7.4 showed that the E_2_ formulation had the highest drug release of 94.91%. Ex vivo skin permeation studies using abdominal male Albino rat skin showed drug release increased up to 97.57% in 8 h for ETD topical gel while the topical niosomal gel only released 81.73% of its entrapped drug within the same time frame. The drug release was faster from the topical gel when compared to the niosomal vesicle. The results showed the potency of niosomal gel formulations for transdermal drug delivery [122]. Alcantar et al. invented and patented a drug delivery system composed of niosomes embedded in a polymer hydrogel for controlled drug release in cancerous tumors. The niosomes were prepared by combining Span 60, cholesterol and dicetyl phosphate in the ratio of 1:1:0.1 and loaded with a fluorescent dye. When immersed in water, the niosomes displayed an exponential dye release initially followed by a steady release [123].

Fetihg prepared non-ionic fluconazole-loaded niosomal gels that can be used in topical ocular drug delivery to treat fungal infections of the cornea. The niosomal gels were prepared using Span 60 and cholesterol, incorporated into poloxamer 407 and chitosan gel formulations. The niosomes that were prepared from 2:1 surfactant:cholesterol (F2) had the highest drug entrapment efficiency of 85.35% and a superior drug release profile. Further increase in the cholesterol content decreased the drug entrapment efficiency possibly due to the regular bilayer structure disruption. F2 niosomal poloxamer gel maintained its appearance and the drug content when stored for eight weeks at 4 °C [23].

Evaluation of the antifungal activity of the F2 niosomal formulation showed that the two tested formulations (F2 poloxamer and F2 chitosan gels) showed good inhibition zones (Table 12). F2 niosomal poloxamer gel and F2 niosomal chitosan gel inhibited both *C. albicans* and *C. tropicum* better than Miconazole, a commercial cream, which was used as a reference. F2 niosomal poloxamer gel had a better minimum inhibition concentration (MIC) on *P. chrysogenum* (MIC = 1.9 µG/mL) when compared to F2 niosomal chitosan gel with MIC of 2.5 µg/mL. Drug transcorneal permeation studies were similar for both gel formulations for 2 h, but F2 poloxamer niosomal gel increased afterwards, possibly due to it being less viscous than F2 chitosan niosomal gel and thus having an accelerated drug flux. The results showed the potential use of cholesterol-based niosomal gel formulations in topical delivery of drugs for the treatment of fungal infections [23].

## 6. Cholesterol-Based Compounds for the Treatment of Dermatological Diseases

The dermal route is used to administer drugs with low melting point, low molecular weight and high lipophilicity. High molecular weight drugs cannot readily penetrate through the stratum corneum and the tight barriers of the epidermis [124]. Dermal drug delivery has attracted some researchers because it is non-invasive and improves patient compliance [125]. The skin needs to be kept healthy since it provides the first line of defense against harmful substances that penetrate into the body [126]. Dermal drug delivery systems is mostly used for the treatment of skin diseases via application of topical formulations [127]. The organized defensive membrane structure of the stratum corneum prevents sufficient drugs penetration via the skin and nanosized drug carriers have been explored to improve the skin penetration of drugs to treat dermatological diseases [128]. This section reviews the use of cholesterol-based compounds in dermatological diseases.

Sandoval et al. reported a case where skin lesions caused by CHILD (Congenital Hemidysplasia, Ichthyosiform erythroderma, Limb Defects) syndrome were successfully treated with topical lovastatin and cholesterol lotion. The 2-month old patient administered the topical formulation containing 2% lovastatin and 2% cholesterol lotion resulted in the complete clearance of the lesions after eight weeks. The cutaneous manifestations of CHILD syndrome show the deficiency of cholesterol and toxic steroid precursor’s accumulation. Since the replacement of cholesterol alone without blocking the metabolite production is not effective, the combination of cholesterol with lovastatin showed improved results [129]. This is because lovastatin inhibits endogenous cholesterol production, thereby preventing the accumulation of toxic metabolites that may disrupt skin differentiation [130].

Khalil et al. reported the administration of topical cream composed of 10–20% glycolic acid with a combination of 2% lovastatin with 2% cholesterol to treat Autosomal Recessive Congenital Ichthyoses (ARCI). The research involved a total of 15 patients (average age 11.2 years, 10 males and 5 females) with ARCI. After two months of administration, a mild response was observed in seven patients, two patients had a good response and six patients showed very good response. After three months, only two patients still had a mild response, two patients showed a good response, nine patients displayed very good response and the last two had an excellent response. Five patients applied the glycolic acid-lovastastin-cholesterol combination on the right side and the lovastatin-cholesterol cream on the left. The right side significantly improved compared to the left side, probably because of the role played by glycolic acid in allowing better skin penetration of the lovastatin-cholesterol cream. The results showed the potential of topical application of glycolic acid, 10–20% cream and a combination of 2% lovastatin and 2% cholesterol in treating patients with ARCI [130].

Atzmony et al. prepared topical cholesterol/lovastatin which was applied by five patients with different types of porokeratosis, two of the patients were suffering from with porokeratosis palmaris et plantaris disseminata (PPPD), one patient with disseminated superficial actinic porokeratosis (DSAP) and two patients with linear porokeratosis (LN). The patients applied the 2% cholesterol/2% lovastatin ointment twice a day for over a period of three months. After three months of treatment with the ointment, the patient with DSAP had only small erythematous macules in the treated areas. The two PPPD patients had decreased scaling and moderately decreased erythema within four weeks of therapy. The two LP patients also had a decrease in scale, erythema and thickness. All the patients showed tolerance to the therapy with no reported irritation, redness or allergic contact dermatitis at the treatment sites. The results showed that topical cholesterol/lovastatin is effective in treating porokeratosis [131].

Ibaraki et al. prepared cationic-, neutral- and anionic-1,2-dioleoyl-sn-glycero-3-phosphocholine (DOPC)/cholesterol-based liposomes and 1,2-dioleoyl-sn-glycero-3-phosphoethanolamine (DOPE)/cholesteryl hemisuccinate (CHEMS) liposomes to investigate the effects of the surface charge and flexibility of liposomes on the dermal drug delivery efficacy. The DOPE/CHEMS liposomes showed a faster transmembrane penetration rate when compared to the DOPC/cholesterol-based liposomes. The skin irritation of the liposomes was determined with a 3D human epidermal skin model. The cationic liposomes induced more skin irritation than the rest of the liposomes, suggesting that neutral or anionic liposomes may be more suitable with the absence of skin irritation [125]. Although the cationic liposomes had a better cellular uptake because of the electrostatic interaction with the negatively charged cell membrane, they exhibited significantly more cytotoxicity than other liposomes, while DOPE/CHEMS liposomes were non-toxic to RAW264.7 cells. The anionic and flexible DOPE/CHEMS liposomes were found to exhibit high intracellular uptake, did not cause skin irritation and displayed high intradermal penetration capacity making them suitable carriers in dermal drug delivery [125].

Mahmoud et al. synthesized cholesterol-PEG decorated gold nanorods (Chol-PEG-GNR) and phospholipid (DSPE)-PEG coated GNR and investigated their preferential accumulation into the human skin layers and their photothermal-based activity on bacteria. Chol-PEG-GNR had a better accumulation in the stratum corneum (~15.5%) than that of DSPE-PEG-GNR (~8.0%) while DSPE-PEG-GNR accumulated better into the skin dermis (~23.5%) than Chol-PEG-GNR (~8.5%). Cholesterol and phospholipid enhanced GNR penetration through the upper layers of the skin due to their effect of modulating cholesterol, fatty acids and cholesteryl esters on the stratum corneum. Chol-PEG-GNR showed a 2-log reduction (99%) in bacterial viable count at MIC value of 0.75 nM, showing good antibacterial activity of the Chol-PEG-SH coating against *S. aureus*, the leading cause of soft tissue and skin infections. Chol-PEG-GNR exhibited photothermal antibacterial activity against *S. aureus* at a sub-MIC of 0.06 nM with the generated localized heat increasing by 35 °C. Thus, Chol-PEG-GNR is a promising nano-therapy for the treatment of skin diseases [132].

Tsai et al. prepared elastic liposomes from cholesterol and Tween 80, which were loaded with naringenin [133]. Naringenin is known as a potent antioxidant for the prevention of oxidative skin damage [134]. The naringenin-loaded elastic liposomes had an average vesicle size between 123.7 and 177.7 nm (except for F3 and F7) and their polydispersity index values ranged from 0.11 to 0.22 (Table 13). In vitro skin permeation studies using rat skin revealed that the permeation rate and cumulative drug release after 24 h were 0.25 ± 0.1 μg/h/cm^3^ and 4.8 ± 2.6 μg/cm^3^ for saturated aqueous solution, 0.37 ± 0.15 μg/h/cm^3^ and 14.4 ± 3.1 μg/cm^3^ for 10% Tween 80 solution and 0.25 ± 0.05~0.76 ± 0.21 μg/h/cm^3^ and 6.4 ± 0.8 ~16.5 ± 3.4 μg/cm for elastic liposomes. The amount of naringenin deposited in stratum corneum, epidermis, dermis and total skin was significantly increased from the elastic liposomes [133].

Tween 80 is an effective penetration enhancer [135], and its presence in the liposomes increased the permeation rate and the cumulative amount of the loaded drug. The elastic liposomes produced enhancement ratios of 1.0–3.0-fold for permeation rate and 1.3–3.5-fold cumulative amount. Formulation F1 displayed the highest enhancement effect. The use of elastic liposomes as drug carrier vesicles had a 1.2–1.9-fold increase on the total deposition amount (19.0 ± 3.7–30.7 ± 13.0 μg/cm^3^) revealing the elastic liposomes are potent systems for topical skin delivery and the transportation of naringenin. The highest total deposition of 30.7 ± 13.7 µg/cm^3^ was observed from naringenin-loaded elastic liposome with medium-level cholesterol and high-level Tween 80. The tested elastic liposomes showed no significant edema and erythema showing their safety as a promising naringenin carrier in topical application [133].

Atopic dermatitis (AD) is an allergic skin disease caused by the skin inflammation and stratum corneum barrier failure [136]. Some small interfering RNA (siRNA) are known to control cytokine production in allergic reactions. Ibaraki et al. developed an siRNA-encapsulated DOPE/CHEMS flexible liposome having AT1002 for effective dermal delivery of siRNA [137]. AT1002 peptide opens the tight junctions of the skin [138] and has been reported to enhance topically applied siRNA delivery. The prepared siRNA-DOPE/CHEMS liposomes had an average size of 70 nm, suitable for dermal application. The siRNA-DOPE/CHEMS liposomes showed higher cellular uptake with no cytotoxicity in RAW264.7 cells and high intradermal penetration in both AD mice skin and the tape-stripped skin. Administration of siRelA-DOPE/CHEMS liposomes plus AT1002 decreased the number of mast cells in AD-induced mice. SiRelA-DOPE/CHEMS liposomes suppressed cytokine production in the ear auricle skin of AD-induced mice, even without the addition of AT1002. The results showed the potential ability of topical application using AT1002 and DOPE/CHEMS liposomes in treating skin diseases such as AD [137].

Jacob et al. prepared and evaluated a noisome gel for enhanced dermal delivery of acyclovir, a potent antiviral drug for herpes simplex viral infections treatment. The niosomes were prepared from different compositions of span 60, Tween 60, cholesterol and lecithin (Table 14). All the niosomes had a particle size of between 24 and 36 µm, entrapment efficiency in the range of 52.43–95.67% and zeta potential values between −37.7 and −42.4 mV. The formulated niosomes enhanced ex vivo drug permeation by ~1.01 to ~4.34 folds with B_8_ niosome gel formulation having the highest permeation of 25.75 µg/cm^2^. Niosome gel formulation B_8_ was studied for ex vivo stratum corneum deposition. After 2 h of application, a greater amount of acyclovir was deposited in the stratum corneum by B_8_ formulation (132.57 µg/cm^2^) compared to that of the commercial acyclovir cream (74.54 µg/cm^2^) (Table 14) [139].

Topical bioavailability studies in rabbits showed that the stratum corneum uptake of acyclovir by B_8_ was high while its clearance after the termination of the therapy was rapid. The formulation accumulated in the dermis or epidermis region of the skin, facilitating drug localization which would enhance the therapeutic efficiency of acyclovir. The formulation did not cause any skin irritation such as erythema and edema in any rabbit. Stability studies showed the vesicles retained ~97.63% of acyclovir at 25 °C when stored for three months. The results suggest that B_8_ formulation can be a viable system for dermal delivery of acyclovir [139].

Wang et al. prepared Paclitaxel (PTX)-cholesterol-loaded-liposomes (PTXL) to improve the water solubility of PTX and investigated its anti-keloid and anti-fibrotic efficacy against keloids. The PTXL average particle size was 101.43 nm, zeta potential of −41.63 mV and polydispersity index (PDI) of 0.244. Entrapment efficiency and loading capacity of PTXL were found to be 95.63% and 2.7%, respectively. When stored for 30 days at 4 °C, PTXL maintained their size, with PDI showing their stability. In vitro drug release showed a sustained release of PTX from PTXL. Cellular uptake studies showed that PTXL were partially taken up by human keloid fibroblasts (HKFs) through caveolin-mediated endocytosis rather than clathrin [140]. PTXL inhibited cell proliferation better than PTX when tested at the same concentration range of 0.05–10 µg/mL. PTXL induced cell apoptosis of HKFs and arrested the HKF cell cycle in G_2_/M phase. Cell invasion results showed that PTXL significantly inhibited the invasion of HKFs with invasion rates of 28.21% and 5.77% for PTX and PTXL, respectively. In vivo results from BALB/c nude mice treated with PTXL, PTX and 5% glucose solution showed that PTXL reduced HKFs density better than both PTX and glucose solution. PTXL inhibited the AKT/GSK3β signaling pathway, thus reducing the production of fibrogenic cytokines such as IL-6, TNF-α and TGF-β, which consequently ameliorated fibrosis progress in keloids. The results showed the potential of PTXL in treating keloids [140].

## 7. Conclusions

Hybrid molecules containing cholesterol have been successfully synthesized in their broad range. These include anticancer, antimicrobials, antimalarials and antivirals. Conjugation of drugs to cholesterol has been achieved by reacting the different functional groups on the drugs, such as carboxylic and amines, with the hydroxyl end of cholesterol. Sometimes a linker is used between cholesterol and the drug. Limitations such as bioavailability and toxicity have been overcome through conjugating drugs to cholesterol. Cholesterol has been used in the design of drug delivery systems for dermal, transdermal, intravenous and oral drug administration. The use of cholesterol in the design of these systems was found to influence the particle size, stability, drug entrapment efficiency, drug release profile and cellular uptake of the systems. Although many positive outcomes have been drawn from the conjugation of therapeutic agents to cholesterol, there is still much work that needs to be done to make sure that they reach all advanced stage in clinical trials.

## Figures and Tables

**Table 1:** Characterization of liposomes with different cholesterol content.

Preparation	Composition (Molar Ratio)	LC (%) of DOX	Diffusivity (µm^2^/s)	Ratio of Diffusivity
Lip1	97.5:-:2.5	10.6	0.14	1
Lip2	77.5:20.0:2.5	10.8	0.75	5.4
Lip3	67.5:30.0:2.2	11.0	1.55	11.1
Lip4	57.5:40.0:2.5	11.1	1.13	8.1
Lip5	47.5:50.0:2.5	1 0.9	0.35	2.5
Lip6	56.3:38.4:5.3	11.0	1.72	12.2

LC (%), drug loading content; -, not applicable. Compositions of the liposomes were HSPC:cholesterol:DSPE-PEG_2000._

**Table 2:** IC_50_ (µg/mL) values for free DOX, free CQ and drug-loaded liposomes on the different cell lines.

	MCF-7	MCF-7/ADR	HL60	HL60/ADR
Free DOX	0.4	15.7	0.4	13.6
Free CQ	18.1	23.3	17.3	11.2
Drug loaded liposomes	0.4	3.3	0.4	1.5

**Table 3:** IC_50_ values of different EPI formulations in S180 and RAW264.7 cell lines.

Cell Line	EPI-S (µM)	EPI-CL (µM)	EPI-PL (µM)	EPI-SAL (µM)
S180	0.48	3.07	11.61	1.41
RAW264.7	0.25	1.11	3.99	0.47

**Table 4:** Encapsulation efficiency and Minimum Inhibition Concentration (MIC) of the three formulations.

Formulation	%EE	MIC (µg/mol)
mPEG-b-PCL-Chol (18)	60	0.46
mPEG-b-PCL	28	0.46
mPEG-Chol	42	0.93
Fungizone	-	0.23

**Table 5:** Sequence and antiviral potency of C34-Chol and controls against HIV-HXB2.

Peptide	Sequence	IC_50_ Viral Infectivity (pM)
C34	WMEWDREINNYTSLIHSLIEESONOOEKNEOELL	205
C34-Chol	WMEWDREINNYTSLIHSLIEESONOOEKNEQELLGSGC (Chol)	270
C34-Aceta	WMEWDREINNYTSLIHSLIEESONOOEKNEQELLGSGC (Aceta)	4
Chol-C34	C(Chol)GSG WMEWDREINNYTSLIHSLIEESONOOEKNEOELL	9515
C34-Pam	WMEWDREINNYTSLIHSLIEESONOOEKNEQELLGSGK (Pam)	713
T20-Chol	YTSLIHSLIEESONOOEKNEOELLELDKWASLWNWFGSG- C (Chol)	3726

**Table 6:** Vesicle types, with mol % of the phospholipid/fatty acid content and average fluorescent increase in response to bacteria.

Vesicle Types and Composition						Average Fluorescent Increment		
Class	Vesicle Types	DPPC	DSPC	Chole-Sterol	TCDA	Hepes (Buffer)	PAO1	MSSA476
A	1	88	-	10	-	37,500	115,000	75,000
	2	78	-	20	-	1000	100,000	37,500
	3	68	-	30	-	1000	37,500	12,500
B	4	73	-	0	25	50,000	105,000	75,000
	5	63	-	10	25	37,500	102,000	76,000
	6	53	-	20	25	12,500	97,000	72,000
	7	43	-	30	25	1000	35,000	69,000
	8	33	-	40	25	1000	13,000	73,000
	9	23	-	50	25	1000	11,000	78,000
C	10	-	88	10	-	500	20,000	10,000
	11	-	78	20	-	550	55,000	11,000
	12	-	68	30	-	200	12,500	5000
D	13	-	73	20	5	500	60,000	35,000
	14	-	68	20	10	450	50,000	40,000
	15	-	63	20	15	500	42,500	62,500
	16	-	58	20	20	550	52,500	85,000
	17	-	53	20	25	400	35,000	37,000
E	18	-	63	10	25	200	20,000	300,000
	19	-	43	30	25	300	25,000	45,000

Hepes buffer was used as the control.

**Table 7:** Mean diameter (MD), zeta potential (ZP), polydispersity index (PDI) and entrapment efficiency (EE %) of the nanohydrogels.

Nanohydrogel	MD (nm)	ZP (mV)	PDI	EE (%)
ESN	554	−30	0.45	-
EAN	383	−24	0.19	-
BSN	507	−25	0.49	38
BAN	341	−23	0.30	35

**Table 8:** Formulations of KTZ niosomes. Ratios (KTZ: surfactant: cholesterol). All ingredients were measure in mg. Ten milliliters of chloroform and 20 mL of PBS (pH 7.4) were added to each formulation.

Formulation Code		IngreDients				
	Ratios	KTZ	Span 40	Span 60	Tween 60	Cholesterol
TNS1	1:1:0.2	100	100	-	-	20
TNS2	1:1.5:0.3	100	150	-	-	30
TNS3	1:2:0.4	100	200	-	-	40
TNS4	1:1:0.2	100	-	100	-	20
TNS5	1:1.5:0.3	100	-	150	-	30
TNS6	1:2:0.4	100	-	200	-	40
TNS7	1:1:0.2	100	-	-	100	20
TNS8	1:1.5:0.3	100	-	-	150	30
TNS9	1:2:0.4	100	-	-	200	40

**Table 9:** Evaluations for KTZ niosomes.

Formulation Code	Particle Size (µm)	% EE	24 h Cumulative Drug Release (%)
TNS1	6.12	55.14	72.371
TNS2	5.76	59.08	68.113
TNS3	5.4	63.02	65.469
TNS4	5.94	69.39	66.068
TNS5	5.22	73.48	59.446
TNS6	4.86	76.96	53.204
TNS7	7.38	71.66	55.909
TNS8	6.84	76.36	49.628
TNS9	6.3	78.63	46.636

**Table 10:** Composition and characterization of lopinavir-loaded niosomal dispersions.

Formulation Code	Lopinavir:Span 40:Cholesterol (Molar Ratio)	Vesicular Size (nm)	PDI	EE (%)
F1	1:0.5:-	354.94	0.228	45.3
F2	1:1.0:-	276.38	0.194	52.9
F3	1:1.5:-	196.45	0.165	58.9
F4	1:2.0:-	203.52	0.216	57.4
F3a	1:1.3:0.2	169.34	0.212	62.5
F3b	1:1.1:0.4	132.86	0.189	68.1
F3c	1:0.9:0.6	105.22	0.127	75.5
F3d	1:0.7:0.8	114.65	0.133	74.5

PDI, polydispersity index.

**Table 11:** Composition molar ratios, entrapment efficiency (EE %) and 24 h cumulative drug release for the different niosomal formulations.

Formulation Code	Drug:Chol:Span 60 (Molar Ratio)	EE (%)	Cumulative Drug Release after 24 h
E_1_	1:0.5:1	85	70
E_2_	1:1:1	96	91
E_3_	1:1.5:1	78	87
E_4_	1:1:0.5	87	82
E_5_	1:1:1.5	89	60

**Table 12:** The antifungal activity of different fluconazole niosomal gel formulations.

Formulation Code	Fungal Strains	F2 Niosomal Poloxamer Gel	F2 Niosomal Chitosan Gel	Miconazole
Inhibition zone (mm)	*C. albicans*	25	18	12
	*C. trolicum*	45	30	25
	*C. chrysogenum*	35	30	0
MIC (µg/mL)	*C. albicans*	15	25	30
	*C. trolicum*	15	21	35
	*C. chrysogenum*	1.9	2.5	Not detected

**Table 13:** Composition and physical characteristics of the naringenin-loaded elastic liposomes.

Code	Cholesterol (mg)	Tween 80 (mg)	Size (nm)	PI	Zeta (mV)	EE (%)
F1	52	68	155.0	0.16	−16.1	99.42
F2	52	52	177.7	0.19	−13.4	99.43
F3	52	34	-			
F4	34	68	123.7	0.16	−11.0	99.52
F5	34	52	138.3	0.13	−12.3	99.56
F6	34	34	176.1	0.22	−15.6	99.53
F7	17	68	-			
F8	17	52	147.3	0.11	−8.2	99.59
F9	17	34	132.3	0.17	−2.2	99.64

Polydispersity index (PI); Encapsulation efficiency (EE).

**Table 14:** Formulation composition, vesicle size, entrapment efficiency (EE%), drug release (%), skin permeation (µg/cm^2^) and enhancement ratio.

Batch No.	Variables in Code		Vesicle Size (µm)	EE %	Drug Release (%)	Skin Permeation (µg/cm^2^)	Enhancement Ratio
	X_1_	X_2_					
B_1_	−1	−1	31.56	60.65	56.32	13.85	2.33
B_2_	−1	0	31.89	65.11	51.27	22.98	3.87
B_3_	−1	1	33.45	65.43	50.75	25.75	4.43
B_4_	0	−1	26.16	53.65	52.27	15.55	2.61
B_5_	0	0	28.41	55.11	54.56	12.81	2.15
B_6_	0	1	35.89	52.43	58.67	18.52	3.11
B_7_	1	−1	23.56	90.73	5.89	6.89	1.16
B_8_	1	0	28.89	95.67	2.89	5.99	1.01
B_9_	1	1	31.45	65.68	14.67	6.19	1.04
Control	-	-	-	-	97.65	5.97	-

Coded values of −1, 0 and 1 stand for X1 ratio of 0.25:0.25, 0.25:0.5 and 0.25:0.75 (mg) and X2 ratio of 1:1, 1:2 and 1:3 (mg). X1 is the ratio of Span 60 to Tween 60 (50:50, 50:100 and 50:150) and X2 is the ratio of cholesterol to lecithin (90:90, 90:180 and 90:270).

## Abbreviations

RNA	Ribonucleic Acid
EDCl	1-Ethyl-3-(3-dimethylaminopropyl)carbodiimide
NHS	N-Hydroxysuccinimide
TEA	Triethylamine
HCl	Hydrochloric Acid
r.t.	room temperature
IC_50_	half maximal inhibitory concentration
DNA	Dioxyribonucleic Acid
THF	Tetrahydrofuran
DSC	Dichlorosilane
DMAP	4-Dimethylaminopyridine
DCM	Dichloromethane
DIC	N,N′-Diisopropylcarbodiimide
DMF	Dimethylformamide
PyBOP	benzotriazol-1-yl-oxytripyrrolidinophosphonium hexafluorophosphate
HOBT	Hydroxybenzotriazole
DIEA	N,N-Diisopropylethylamine
CLSM	Confocal Laser Scanning Microscopy
DMSO	Dimethyl sulfoxide
MDR	Multi Drug Resistance
MTT	3-(4,5-dimethylthiazol-2-yl)-2,5-diphenyltetrazoliumbromide
